# Shared genetic factors and the interactions with fresh fruit intake contributes to four types squamous cell carcinomas

**DOI:** 10.1371/journal.pone.0316087

**Published:** 2024-12-31

**Authors:** Mengqing Mo, Can Hou, Huangbo Yuan, Renjia Zhao, Mingyang Chen, Yanfeng Jiang, Kelin Xu, Tiejun Zhang, Xingdong Chen, Chen Suo

**Affiliations:** 1 Department of Epidemiology & Ministry of Education Key Laboratory of Public Health Safety, School of Public Health, Fudan University, Shanghai, China; 2 Department of Outpatient Office, Shanghai Skin Disease Hospital, School of Medicine, Tongji University, Shanghai, China; 3 West China Biomedical Big Data Center, West China Hospital, Sichuan University, Chengdu, China; 4 State Key Laboratory of Genetic Engineering, Zhangjiang Fudan International Innovation Center, Human Phenome Institute, Fudan University, Shanghai, China; 5 Fudan University Taizhou Institute of Health Sciences, Taizhou, Jiangsu, China; 6 Department of Biostatistics, School of Public Health, Fudan University, Shanghai, China; 7 Shanghai Institute of Infectious Disease and Biosecurity, Shanghai, China; 8 National Clinical Research Center for Aging and Medicine, Huashan Hospital, Fudan University, Shanghai, China; 9 Yiwu Research Institute of Fudan University, Yiwu, Zhejiang, China; The First Hospital of Jilin University, CHINA

## Abstract

Studies have reported risk factors for a single-squamous cell carcinoma(Single-SCCs). However, the shared common germline genetic factors and environmental factors have not been well elucidated with respect to augmented risk of pan-squamous cell carcinoma(Pan-SCCs). By integrating a large-scale genotype data of 1,928 Pan-SCCs cases and 7,712 age- and sex-matched controls in the UK Biobank cohort, as well as multiple transcriptome and protein databases, we conducted a multi-omics analysis. Genome-wide association analysis (GWAS) was used to identify genetic susceptibility loci of SCCs. High resolution human leucocyte antigen (HLA) alleles and corresponding amino acid sequences were imputed using SNP2HLA and tested for association with SCCs. Credible risk variants (CRVs) were combined risk SNPs reported in GWAS Catalog and our study, followed by comprehensive bioinformatics analyses. We identified six novel index SNPs in the progression of SCCs, which were also strongly interacted with fresh fruit intake. Moreover, our study systematically characterize the HLA variants and their relationship to SCCs susceptibility. We identified HLA-A*01 and six HLA-A amino acid position were associated independently with SCCs. Credible risk variants were annotated to 469 target genes, further GO and KEGG Pathway Enrichment Analysis showed that SCCs genes were primarily involved in immune-related pathways, espechially regulated by HLA region. The transcriptome analysis showed that there were 270 differentially expressed genes(DEGs), with the upregulated genes were enriched in the regulation of stem cell differentiation, proliferation, development, and maintenance. The PPI Network and Modular Analysis uncovered the Keratin(KRT) genes may serve as a potential marker in SCCs. Our results illustrate the molecular basis of both well-studied and new susceptibility loci of SCCs, providing not only novel insights into the genetic commonality among SCCs but also a set of plausible gene targets for post-GWAS functional experiments.

## Introduction

Squamous cell carcinomas (SCCs) represent the most prevalent type of solid tumors, originating primarily from the epithelial tissues of either the aerodigestive or genitourinary tracts. Common sites of occurrence include the head and neck, esophagus, lung, and cervix [1]. The burden of these cancers is globally significant. For instance, the International Agency for Research on Cancer (IARC) highlighted in their 2018 report that cervical cancer, predominantly caused by SCCs, ranks as the fourth most common cancer among women worldwide [2]. Similarly, the incidence and mortality rate of esophageal cancer, another common site for SCCs, are alarmingly high, ranking 6th and 4th respectively on a global scale [3]. This widespread prevalence of SCCs across various sites contributes significantly to the global cancer burden, accounting for more than 2 million new cases and 1.5 million deaths annually [4]. Furthermore, the highly aggressive nature of SCCs leads to a high recurrence rate and results in a a 5-year survival rate of less than 20% [5]. Consequently, the impact of SCCs on human health is profound and alarming.

Generally speaking, research into the risk factors, preventions and treatments of SCCs follows the anatomical divisions of clinical medicine. However, substantial evidence suggests a commonality in determinants across SCCs, regardless of their anatomical location, indicating a unified disease spectrum [1]. The pathogenesis of SCCs involves a multi-step process marked by the accumulation of genetic mutations, leading to the generation of preneoplastic lesions that progress into invasive carcinomas [6]. Common factors influence the process of squamous differentiation at various sites. For example, shared environmental risk factors include infection, carcinogens, drugs and radiation [1]. Additionally, histological similarities, notably the formation of keratin pearls indicative of squamous differentiation, are observed across different SCC sites [7]. Moreover, there is a notable overlap in key risk genes and pathways implicated in SCC development [5]. Studies, including those from The Cancer Genome Atlas (TCGA), reveal a consistent mutational landscape in SCCs across different anatomical sites, showing that tumors with similar pathologic classifications tend to cluster together [8]. SCCs also display molecular signatures distinct from other types of cancer, with immune-signaling subtypes indicating the relationship between histopathology and immune infiltration types [9]. Analysis of SCC tumors from 5 origins (lung squamous cell carcinoma, head and neck squamous cell carcinoma, cervical squamous cell carcinoma, esophageal carcinoma, and bladder carcinoma) revealed a tighter co-localization on TumorMap and integration into three major clusters, distinguishing them from other cancers. These findings underscores the influence of the cell of origin on the cancer’s molecular patterns and lend further support to the concept of a pan-squamous sub-analysis.

Previous studies have extensively examined the risk factors associated with SCCs, with however a focus on the tumor cell-intrinsic characteristics of SCCs occurring at only a specific site (i.e., single-SCC) [10]. In contrast, research on the joint influence of genetic and environmental factors in predicting Pan-SCC, which involve SCCs at two or more sites, has been limited. By taking Pan-SCCs into consideration, with a focus on their histological similarities, there is a potential to uncover additional genetic factors that may have been overlooked in single-SCC studies. Additionally, the role of Human Leukocyte Antigen (HLA) genes in SCC progression—critical for modulating antigen presentation and immune response—has been insufficiently explored [11–15]. Further, research highlights that molecular gene expression profiles, indicative of early carcinogenesis, can forecast disease progression, underlining the importance of identifying differentially expressed genes (DEGs) for precise SCC diagnosis and treatment [16, 17]. Therefore, in this study, our objective is to assess genetic risk factors for Pan-SCCs, with a focus on HLA genes, DEGs, and gene-environment interactions, aiming to bridge these knowledge gaps.

## Materials and methods

### Study design

**Fig 1:**
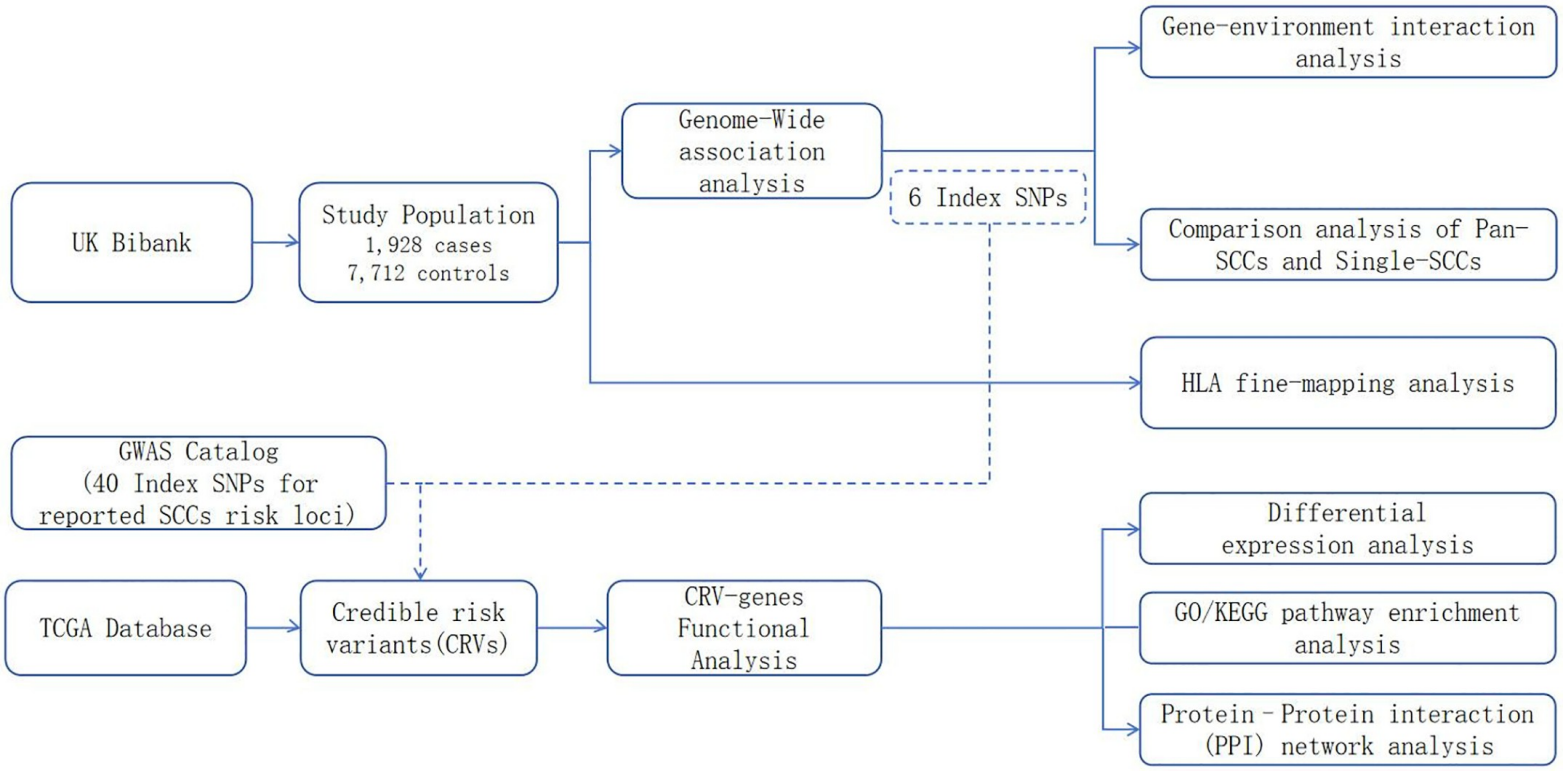
Components of the study design. The study includes two components: (1) using data from UKB to reveal shared susceptibility variants of SCCs and the interactions with fresh fruit intake; (2) combining GWAS Catalog and TCGA transcriptome data to determine shared risk genes or pathways of SCCs.

Fig 1 illustrates the detailed design of our study. Initially, leveraging data from the UK Biobank, we conducted a genome-wide association study (GWAS) involving 1,928 cases of SCCs and 7,712 control subjects. We also conducted a Gene-Environment Interaction analysis to investigate the interplay between genetic risk variants and lifestyles factors (smoking, alcohol consumption, and the intake of vegetables and fresh fruits). The statistically significant variants identified in our GWAS were then amalgamated with previously identified genome-wide significance variants from the GWAS catalog database, resulting in a curated set of credible risk variants (CRVs). Moreover, a detailed HLA fine-mapping analysis was conducted to investigate HLA SNPs and specific amino acid polymorphisms within the human leukocyte antigen region. Then, functional implications of CRVs on gene expression were elucidated to identify CRV-associated genes. Finally, we conducted functional analyses, included pathway enrichment analysis, differential expression analysis, and protein—protein interaction network analysis, to explore potential risk pathways and biological mechanisms of SCCs.

### Data source

Part of this research drew from the UK Biobank database, a large-scale biomedical database contains medical records of 502,368 participants aged between 37 and 73 years, who were enrolled from 2006 to 2010. The database provided extensive health-related information through baseline or follow-up online questionnaires, verbal interviews, biological samples, and physical assessments. Approval for this study was granted by the North West Multi-Center Research Ethics Committee, with approval numbers: 11/NW/0382, 16/NW/0274, and 21/NW/0157, and written informed consent was obtained from each participant.

### GWAS of Pan-SCCs in the UK Biobank

We utilized data from the UK Biobank (UKB), a large prospective cohort study that enrolled over 0.5 million participants aged between 40 to 69 from 2006 to 2010 [18]. The UKB’s extensive collection of phenotypic and genomic data provides a unique resource for investigating the genetic underpinnings of SCCs. We implemented comprehensive quality control measures using PLINK to exclude ineligible individuals and variants [19]. Based on the imputed genotype data from the UKB, we excluded individuals with missing genotype data exceeding 2%, those with discrepancies between reported and genetically inferred sex, and individuals identified as outliers based on ancestry or genetic relatedness, ensuring a homogenous study population of unrelated individuals (beyond second-degree relatives) with Caucasian ethnicity. Variants were filtered out based on the following criteria: minor allele frequency (MAF) less than 1%, call rate below 98%, and deviations from Hardy-Weinberg equilibrium (p-value<1e-6). Following the rigorous QC process, we conducted a nested case-control GWAS study on 1,928 SCCs cases, including 802 head and neck squamous cell carcinoma (HNSCC), 632 lung squamous cell carcinoma (LSCC), 315 cervical squamous cell carcinoma (CESC), and 179 esophageal squamous cell carcinoma (ESCC), along with 7712 controls without any cancer diagnosis. SCCs cases were ascertained based on the International Classification of Diseases, Tenth Revision (ICD-10) and histological coding (see S1 Table). The GWAS was performed using logistic regression model, adjusting for age, sex and the first ten principal components of genetic ancestry to control for population stratification.

### Gene-environment interaction analysis

Gene-environment interactions were comprehensively investigated on a multiplicative scale. Type 2 gene-environment interaction (GEI) model were established, so as to assess the interactive contribution of SNPs and environmental factors to SCCs risk [20]. We assessed p-adj for multiplicative interactions through the inclusion of a product interaction term. The baseline for comparison was defined as individuals without genetic susceptibilities and without unhealthy lifestyle behaviors. The analysis aimed to compare the impact of each specific combination of genetic and lifestyle factors against this baseline group. An interaction is deemed significant when the combined influence of having a risk genotype and unhealthy lifestyle behaviors markedly differs from the effects of these factors when considered separately. Statistical significance was identified in case of p-adj < 0.05 after multiple comparison adjusted.

### Identification of the credible risk variants

In our analysis, variants were deemed significant if they achieved a GWAS p-value of less than 5e-7. From this, we established a comprehensive set of risk-associated index variants for Pan-SCCs, incorporating both 357 previously identified SNPs through GWAS catalog database searching and those achieving genome-wide significance in the current GWAS. For the 357 previously reported variants, we defined index SNP based on two key criteria: (1) MAF ≥0.01; and (2) the SNPs linkage disequilibrium (LD, r^2^ <0.6). Similarly, for newly identified risk variants in our study, index variants were categorized by the above same criteria. Consequently, this approach led to the identification of 46 index variants derived from both the 357 previously reported SNPs and those discovered in the current GWAS. Further exploration involved mining for SNPs in strong LD (r^2^ ≥0.6) with the defined index SNPs, positioned within a 500 kb range either upstream or downstream of the index SNP.. All identified index SNPs, along with the associated SNPs in strong LD, were categorized as credible risk variants (CRVs), totaling 643 variants.

### HLA fine-mapping analysis

We performed imputation of classical HLA alleles and amino-acid polymorphisms for both class I (HLA-A, -B, -C) and class II (-DPA1, -DPB1, -DQA1, -DQB1, and -DRB1) loci using the SNP2HLA software and a reference panel composed of individuals of European descent [21]. In total, Our analysis encompassed 4,435 SNPs, 468 amino-acid changes and 62 classical HLA alleles with MAF>0.01 and variants with an information score>0.9. Then, we used logistic regression model to assess the association between the allelic dosages of all imputed variants and risk of SCCs.

### Pathway enrichment analysis

For CRV identified, we employed the ANNOVAR software to annotate them with their nearest gene, resulting in a comprehensive list of genes link with CRVs (CRV-genes) [22]. We then performed Gene Ontology (GO) and Kyoto Encyclopedia of Genes and Genomes (KEGG) pathway enrichment analyses on the CRV-genes to elucidate their potential roles in the etiology of SCCs. Enrichment analyses were conducted using the R package clusterProfiler(version 4.1.0), with the threshold for significance set at a false discovery rate-adjusted p-value (q-value) of less than 0.05.

### Differential expression analysis based on TCGA data

To identify differentially expressed CRV-genes between tumor and adjacent normal tissues, we analyzed RNA-seq data of patients diagnosed with CESC, ESCA, HNSCC, and LUSC, from The Cancer Genome Atlas (TCGA) database. Differentially expressed genes (DEGs) were analyzed using the R package DESeq2(version 4.1.0), with a threshold set at |log2(fold change)| ≥ 1 and p-adj < 0.05. The P value was adjusted for the six multiple comparisons by Bonferroni test.

### Protein—Protein Interaction (PPI) network, clustering, and visualization

All identified DEGs were aggregated and underwent PPI functional enrichment analysis using STRING (version 11.5) to construct the PPI network [23]. Results from STRING were imported into Cytoscape software (version 3.8.0) for the visualization of the molecular interaction networks and integration of the gene expression profiles of the DEGs [24]. Further analysis of the target network and protein clustering was performed using the Cytoscape MCODE plugin (parameter: degree cut-off = 2, node score cut-off = 0.2, node density cut-off = 0.1, K-score = 4, and max depth = 100).

### Statistical analysis

R software was employed to perform the statistical analyses. Chi-square test was applied to compare categorical data for population characteristics in cases and controls. Univariate logistic regression was implemented to evaluate the association of each population characteristic with SCCs risk.

### Ethics approval and consent to participate

UK Biobank was approved by the North West Multi-Centre Research Ethics Committee (Ref: 11/NW/0382), and all participants provided written informed consent to participate in the UK Biobank study. The study protocol is available online (http://www.ukbiobank.ac.uk/). Open squamous cell carcinomas gene expression datasets were downloaded from The Cancer Genome Atlas (TCGA) databases.

## Results

### Characteristics of the study population

We included a total of 1,928 individuals diagnosed with SCCs (including 802 HNSCC cases, 632 LSCC case, 315 CESC cases and 179 ESCC cases), alongside 7,712 healthy controls from the UK Biobank. Demographic and lifestyle characteristics for the SCC patients and healthy controls are shown in Table 1. The average age across both groups was approximately 60 years, with nearly half of the participants being female. Compared with healthy controls, SCC patients were more likely to smoke and consume alcohol, and less likely to have enough vegetable intake and fruit intake. As shown in Table 2, education level, smoking history and status, alcohol consumption status, and intake of vegetables and fruits were all significantly associated with risk of SCCs.

**Table 1:** Distributions of population characteristics between cases and controls.

Variables	Case(N = 1,928)	Control(N = 7,712)	P
**Sex**			/
*female*	901(46.73%)	3604(46.73%)	
*male*	1027(53,27%)	4108(53,27%)	
**Age(mean±sd)**	57.977±8.416	57.977±8.416	/
**Smoking history**			2.2×10^−16^
*no*	383(19.97%)	2980(38.82%)	
*yes*	1535(80.03%)	4696(61.18%)	
**Smoking status**			2.2×10^−16^
*never*	542(28.26%)	4024(52.41%)	
*previous*	823(42.91%)	2900(37.78%)	
*current*	553(28.83%)	753(9.81%)	
**Alcohol status**			1.1×10^−15^
*never*	59(3.06%)	265(3.44%)	
*previous*	150(7.78%)	262(3.40%)	
*current*	1718(89.16%)	7182(93.16%)	
**Cooked vegetables**			4.2×10^−10^
= *0*	98(5.29%)	200(2.66%)	
>*0*	1753(94.71%)	7309(97.34%)	
**Raw vegetables**			6.8×10^−14^
= *0*	314(17.39%)	778(10.73%)	
>*0*	1492(82.61%)	6473(89.27%)	
**Fresh fruit**			2.2×10^−16^
= *0*	298(16.09%)	435(5.83%)	
>*0*	1554(83.91%)	7023(94.17%)	

Values are mean ± SD or n(%); The P value was obtained by chi-square tests.

**Table 2:** Associations of population characteristics and SCCs.

Variables	OR	95%CI	P
**Smoking status**			
*no*	1.00		
*yes*	1.78	(1.60, 1.99)	**<0.001**
**Smoking history**			
*never*	1.00		
*previous*	1.63	(1.46, 1.83)	**<0.001**
*current*	2.89	(2.49, 3.34)	**<0.001**
**Alcohol status**			
*never*	1.00		
*previous*	1.77	(1.27, 2.49)	**<0.001**
*current*	1.18	(0.93, 1.52)	0.18
**Cooked vegetables**			
= *0*	1.00		
>*0*	0.88	(0.84, 0.92)	**<0.001**
**Raw vegetables**			
= *0*	1.00		
>*0*	0.91	(0.88, 0.93)	**<0.001**
**Fresh fruit**			
= *0*	1.00		
>*0*	0.80	(0.78, 0.82)	**<0.001**

OR = Odds Ratio; 95% CI = 95% Confidence Interval; The P value was obtained by univariate logistic regression model.

### Genome-wide association analysis of Pan-SCCs

**Fig 2:**
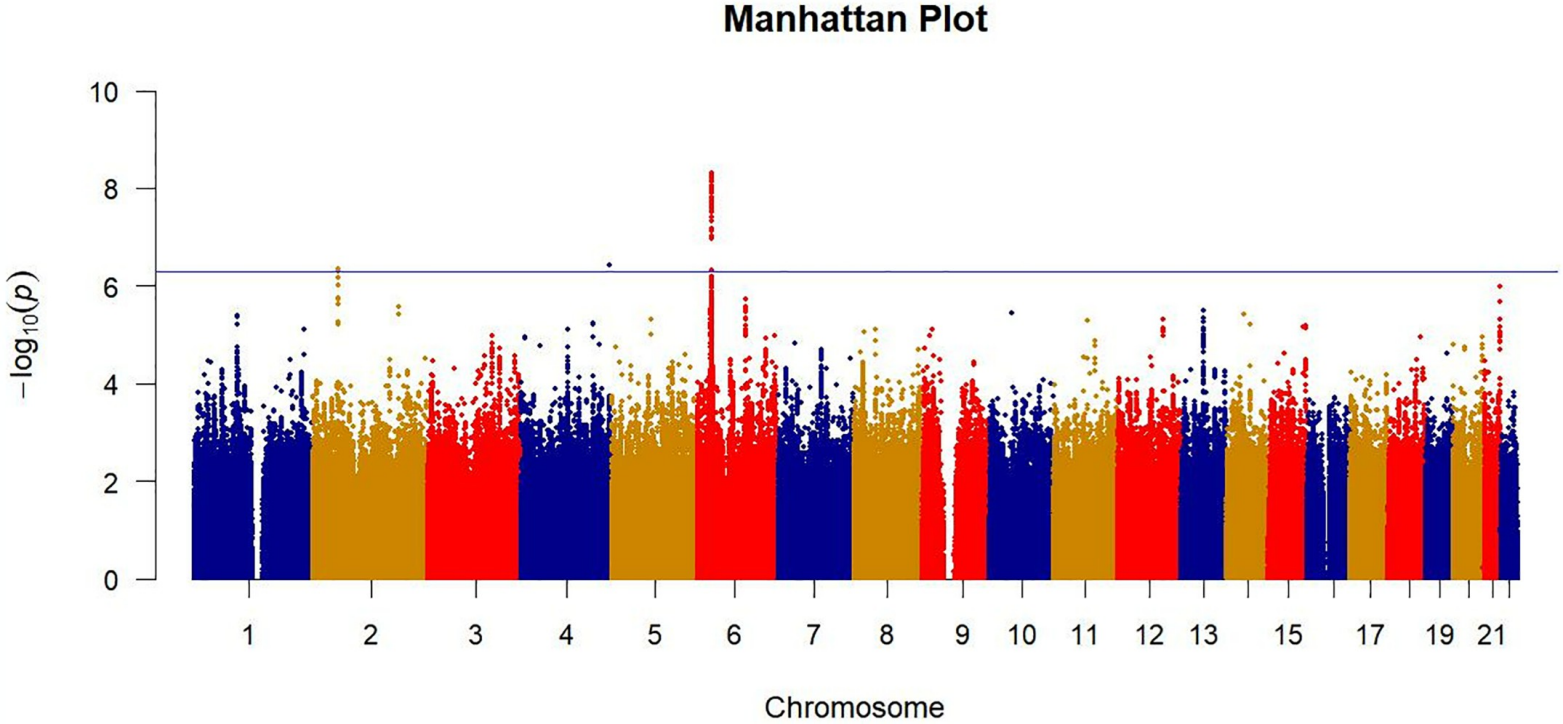
Manhattan plot showing the genome-wide P-values of association. The genome-wide P-values in 1,928 cases and 7,712 controls from UKB are shown. The blue horizontal line represents the threshold of P = 5.0 × 10^−7^, totally 93 significant SNPs.

**Fig 3:**
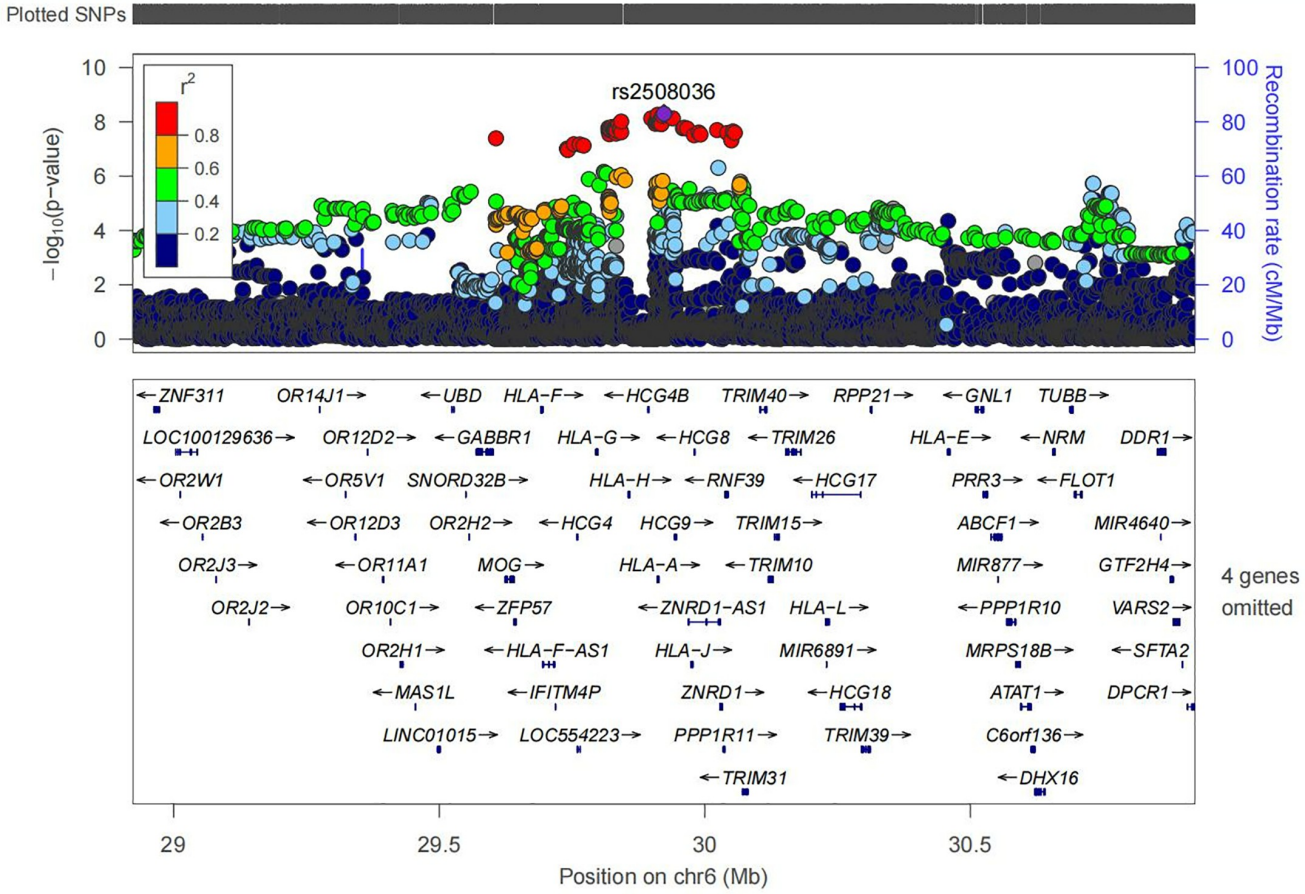
Regional locus zoom plot of the index SNP(rs2508036). The SNPs surrounding rs2508036 are color coded to reflect their correlation with rs2508036. Each dot is colored by r2 of linkage disequilibrium (LD) with the purple-colored index SNP indicated with texts (chromosome position). Genes, the position of exons and direction of transcription from UCSC genome browser are noted. Plots were generated using LocusZoom.

GWAS identified a total of 93 SNPs with p-values <5×10^−7^ (See Fig 2 and S2 Table). The genomic inflation factor (i.e., λ_GC_) was estimated to be 1.032, suggesting no systematic inflation in the GWAS (See S1 Fig). These significant SNPs were predominantly located across three genomic regions on chromosomes 2, 4, and 6, with most showing high LD with each other. We identified six independent index SNPs among them, with rs2508036 demonstrating the most significant association (chr6:29923008, OR = 1.29, 95%CI 1.19–1.41, Table 3). Further annotation of index SNPs within ±500 kb upstream and downstream regions identified nearby functional genes (See S2 Fig). Notably, four index SNPs were located within the HLA region on chromosomes 6, where SNP rs2508036 was in close proximity to HLA-A (Fig 3). Additionally, the relationship between the six index SNPs and SCCs was evaluated under genetic models: codominant, dominant, and recessive. Univariate analysis revealed that all the index SNPs were associated with an increased risk of SCCs across the three models, with homozygotes showing a higher risk than heterozygotes (see S3 Table).

**Table 3:** Functional annotation of the index SNPs.

Index SNP	Chrom	BP	REF	ALT	MAF			OR	95%CI	P	Gene	Consequence
					Case	Control	All					
rs10164641	2	55123380	T	C	0.454	0.4082	0.4173	1.20	1.12–1.29	4.57×10^−7^	EML6	intron_variant
rs80337402	4	18507935	T	C	0.02804	0.01553	0.01802	1.83	1.45–2.32	3.68×10^−7^	ENPP6	intron_variant
rs1264712	6	30052942	G	A	0.2369	0.1952	0.2035	1.28	1.17–1.39	2.24×10^−8^	TRIM31 RNF39	intron_variant
rs3095268	6	29606761	G	A	0.2194	0.1795	0.1875	1.28	1.17–1.39	4.03×10^−8^	SUMO2P1	upstream_gene_variant
rs2508036	6	29923008	G	C	0.2286	0.1853	0.194	1.29	1.19–1.41	5.02×10^−9^	HLA-A HLA-W	intergenic_variant upstream_gene_variant
rs1611673	6	29825415	G	A	0.2287	0.1872	0.1955	1.28	1.18–1.40	1.56×10^−8^	HCG4P7 MICF	intergenic_variant

CHR = Chromosome Number; BP = Chromosome Coordinates (the reference genome is GRCh37/hg19); REF = Reference Allele; ALT = Effect Allele; MAF = Minor Allele Frequency; OR = Odds Ratio; 95% CI = 95% Confidence Interval; The P and the OR was calculated for each SNP in the logistic regression model by adjusting age, sex, and the top 10 genetic principal components.

### Associations between index SNPs and fresh fruit intake and their interaction in Pan-SCCs susceptibility

Gene-environment interactions analysis revealed significant multiplicative interactions for all six index SNPs with fresh fruit intake found (Table 4). Specifically, for individuals carrying rs3095268 GG genotypes, the OR for those not consuming fresh fruit was 2.65. In contrast, the OR for carrying either rs3095268 AG or AA genotypes alone was 1.39, while the interaction effect was 2.89, This indicates that presence of both no fresh fruit intake and rs3095268 AG+AA genotypes simultaneously results in an secondary multiplicative interaction. Similarly, the interactions between other three SNPs and fresh fruit intake were followed secondary multiplicative models, including rs1264712, rs2508036 and rs1611673.

**Table 4:** Effect of fresh fruit intake, index SNPs genotype and diet-gene interaction on SCCs.

Fresh fruit intake	Genotype	Case(n)	Control(n)	OR	95%CI	β	p-adj
**rs10164641**							0.264
*yes*	*TT*	463	2383	1.00			
*yes*	*CT+CC*	1044	4472	1.20(ORg1)	1.07~1.36	0.18(βg1)	
*no*	*TT*	96	241	2.05(ORe1)	1.58~2.64	0.72(βe1)	
*no*	*CT+CC*	248	403	3.17(ORe1g1)	2.63~3.82	1.15(βe1g1)	
**rs1264712**							0.044
*yes*	*GG*	864	4455	1.00			
*yes*	*GA+AA*	644	2392	1.39(ORg1)	1.24~1.55	0.33(βg1)	
*no*	*GG*	211	410	2.65(ORe1)	2.21~3.18	0.98(βe1)	
*no*	*GA+AA*	132	233	2.92(ORe1g1)	2.33~3.65	1.07(βe1g1)	
**rs80337402**							0.799
*yes*	*TT*	1429	6657	1.00			
*yes*	*CT+CC*	82	208	1.84(ORg1)	1.41~2.38	0.61(βg1)	
*no*	*TT*	321	623	2.40(ORe1)	2.07~2.78	0.88(βe1)	
*no*	*CT+CC*	23	22	4.87(ORe1g1)	2.70~8.81	1.58(βe1g1)	
**rs3095268**							0.024
*yes*	*GG*	909	4635	1.00			
*yes*	*AG+AA*	599	2201	1.39(ORg1)	1.24~1.56	0.33(βg1)	
*no*	*GG*	221	426	2.65(ORe1)	2.21~3.16	0.97(βe1)	
*no*	*AG+AA*	123	217	2.89(ORe1g1)	2.29~3.64	1.06(βe1g1)	
**rs2508036**							0.033
*yes*	*GG*	883	4539	1.00			
*yes*	*GC+CC*	612	2254	1.40(ORg1)	1.24~1.57	0.33(βg1)	
*no*	*GG*	213	420	2.61(ORe1)	2.17~3.12	0.96(βe1)	
*no*	*GC+CC*	130	221	3.02(ORe1g1)	2.40~3.79	1.11(βe1g1)	
**rs1611673**							0.028
*yes*	*AA*	890	4554	1.00			
*yes*	*AG+GG*	618	2300	1.37(ORg1)	1.23~1.54	0.32(βg1)	
*no*	*AA*	214	418	2.19(ORe1)	2.17~3.13	0.96(βe1)	
*no*	*AG+GG*	130	225	2.96(ORe1g1)	2.35~3.71	1.08(βe1g1)	

OR = Odds Ratio; 95% CI = 95% Confidence Interval; OReg = OReORg is a multiplicative model; OReg > OReORg is a super multiplicative model; OReg < OReORg is a secondary multiplicative model; OReg = OReORg-1 is an additive model. OReg represents the OR carrying both the risk genotype and risk environment factors, ORe represents the OR of the risk environment factors alone, and ORg represents the OR carrying risk genotype alone. P-adj is adjusted for the six multiple comparisons by Bonferroni test.

### GO and KEGG pathway enrichment analysis

**Fig 4:**
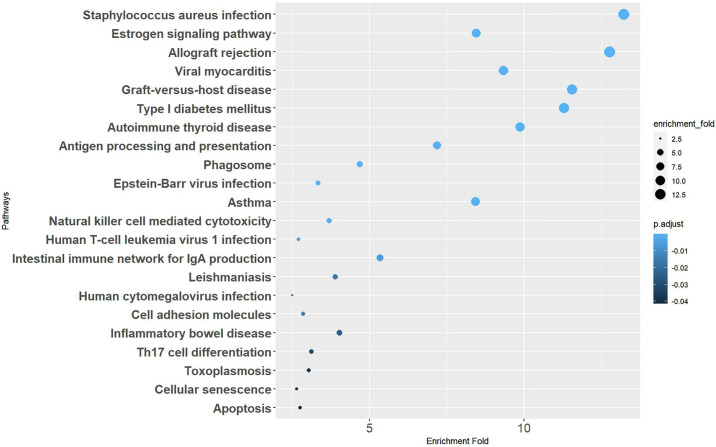
KEGG pathway enrichment analysis. Bubble diagram showing the signaling pathways enriched through Kyoto Encyclopedia of Genes and Genomes (KEGG) analysis of CRV-Genes.

**Fig 5:**
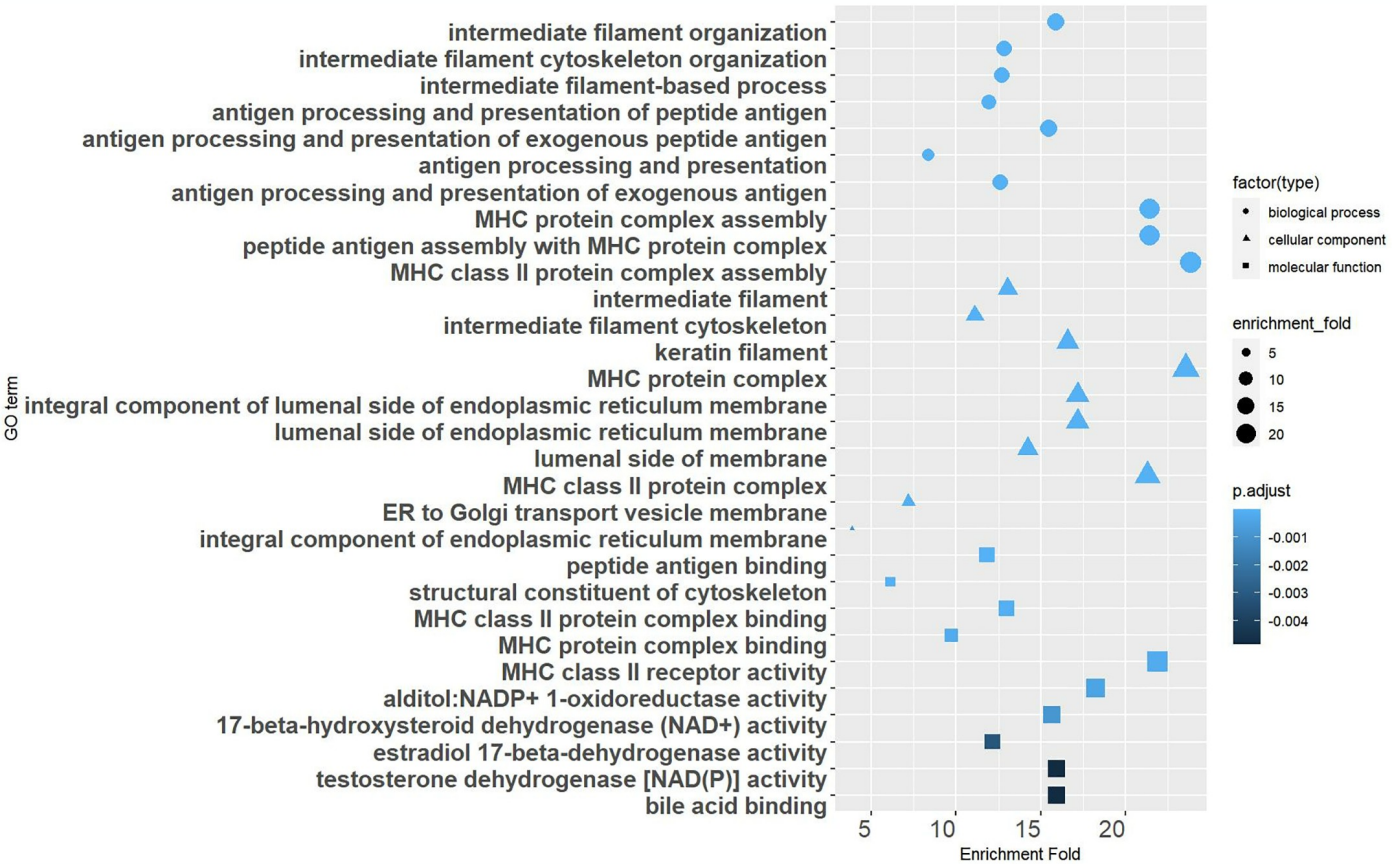
GO functional enrichment analysis. Bubble diagram showing the GO terms enriched through Gene Ontology (GO) analysis of CRV-Genes.

To gain further insight into the identified CRV-genes, pathway enrichment analyses were conducted using the KEGG database. KEGG enrichment analyses revealed that a variety of complex signaling pathways play significant roles in the development of SCCs. Notably, most of the enriched pathways are associated with immune-related functions, such as antigen processing and presentation, natural killer cell mediated cytotoxicity, and immune cell differentiation (Fig 4). GO enrichment analysis further highlighted some biological processes implicated in SCCs, primarily encompassing antigen processing and presentation, MHC protein complex assembly, and intermediate filaments functions, with the first two processes closely related to immune responses. As for cellular component and molecular function, the findings predominantly pertained to the human MHC region, underscoring the significance of immune system involvement in SCC pathogenesis (Fig 5).

### HLA analysis

Following rigorous quality control measures that excluded variants with a low frequency (MAF<0.01) and poor imputation accuracy (information score<0.9), the analysis included 86 classical HLA alleles and amino acids variants. The HLA haplotype demonstrating the most significant association with SCCs was HLA-A*01(P = 2.85×10^−8^, OR = 1.27, 95% CI 1.17–1.40), including a specific association with HLA-A*01:01 (P = 2.85×10^−8^). Given the high level of LD in the HLA gene region, we further conducted conditional analysis to investigate the independent effects of this HLA allele. Conditional analyses confirmed that HLA-A*01 was independently associated with SCCs. Furthermore, the SNP2HLA imputed amino acid positions in the HLA region, identifying six amino acid position significantly associated with SCC risk, all encoded by HLA-A. Notably, amino acid position 67 (P = 2.80×10^−8^, OR = 1.28, 95% CI 1.17–1.40, Table 5) exhibited a stronger association with SCC risk than single classic HLA allele (OR = 1.27).

**Table 5:** The strongest HLA amino acid associations of SCCs.

Chrom	Gene	Variant	OR(95%CI)	P
6	HLA-A	**Position 67**		2.80×10^−8^
		*Valine*	1.00	
		*Methionine*	1.28(1.17,1.40)	
6	HLA-A	**Position 150**		2.85×10^−8^
		*Alanine*	1.00	
		*Valine*	1.27(1.17,1.40)	
6	HLA-A	**Position 158**		2.85×10^−8^
		*Alanine*	1.00	
		*Valine*	1.27(1.17,1.40)	
6	HLA-A	**Position 44**	1.00	2.98×10^−8^
		*Arginine*		
		*Lysine*	1.27(1.17,1.40)	
6	HLA-A	**Position 152**		8.77×10^−6^
		*Glutamic acid*	1.00	
		*Valine*	1.20(1.11,1.30)	
6	HLA-A	**Position 163**		9.18×10^−6^
		*Threonine*	1.00	
		*Arginine*	1.18(1.10,1.27)	

OR = Odds Ratio; 95% CI = 95% Confidence Interval; The P and the OR was calculated for each variant in the logistic regression model, by adjusting age, sex, and the top 10 genetic principal components.

### Identification of DEGs via Protein—Protein Interaction (PPI) network and modular analysis

**Fig 6:**
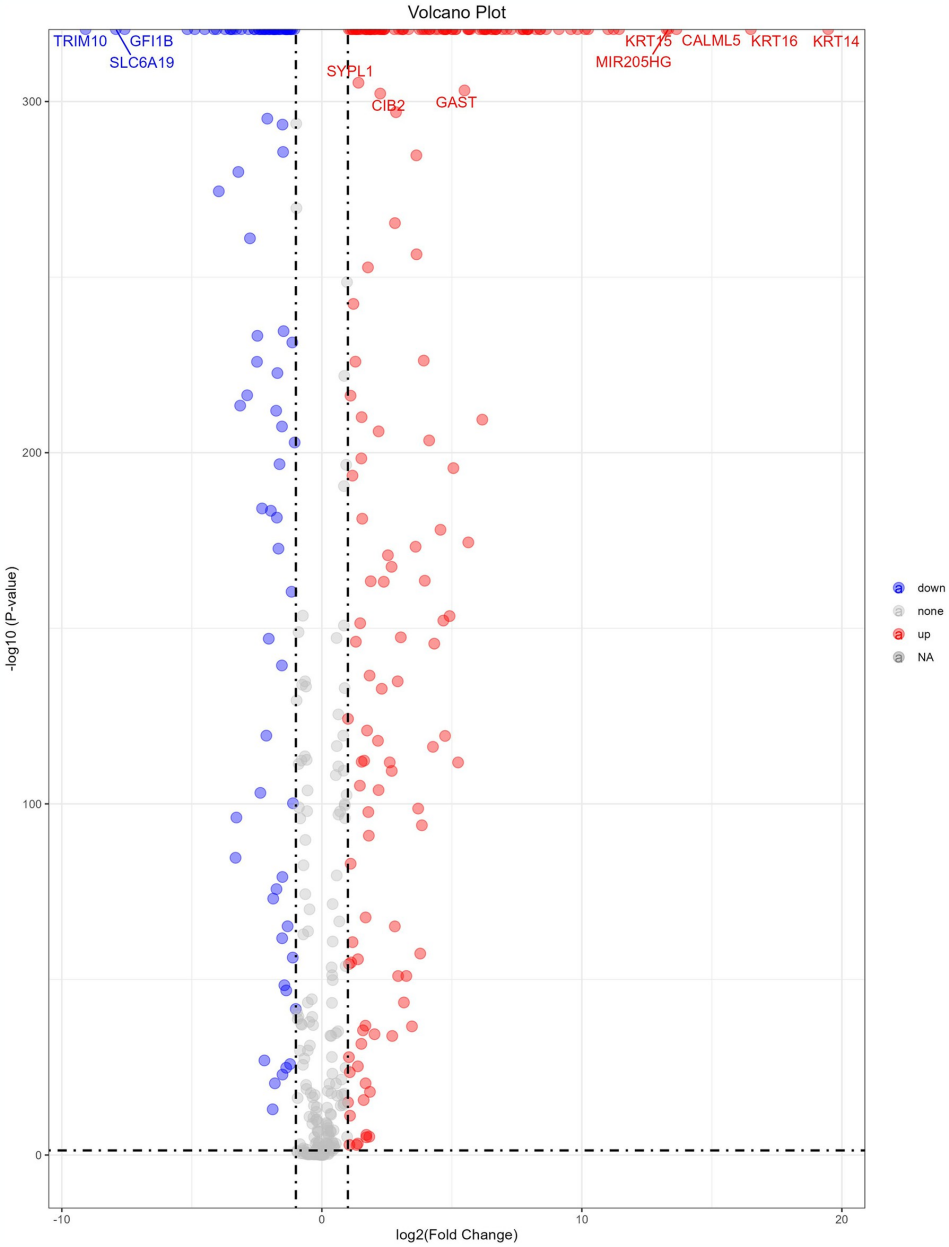
Volcano plot of DEGs. Volcano plot showing the DEGs between the SCCs cases of healthy controls.

**Fig 7:**
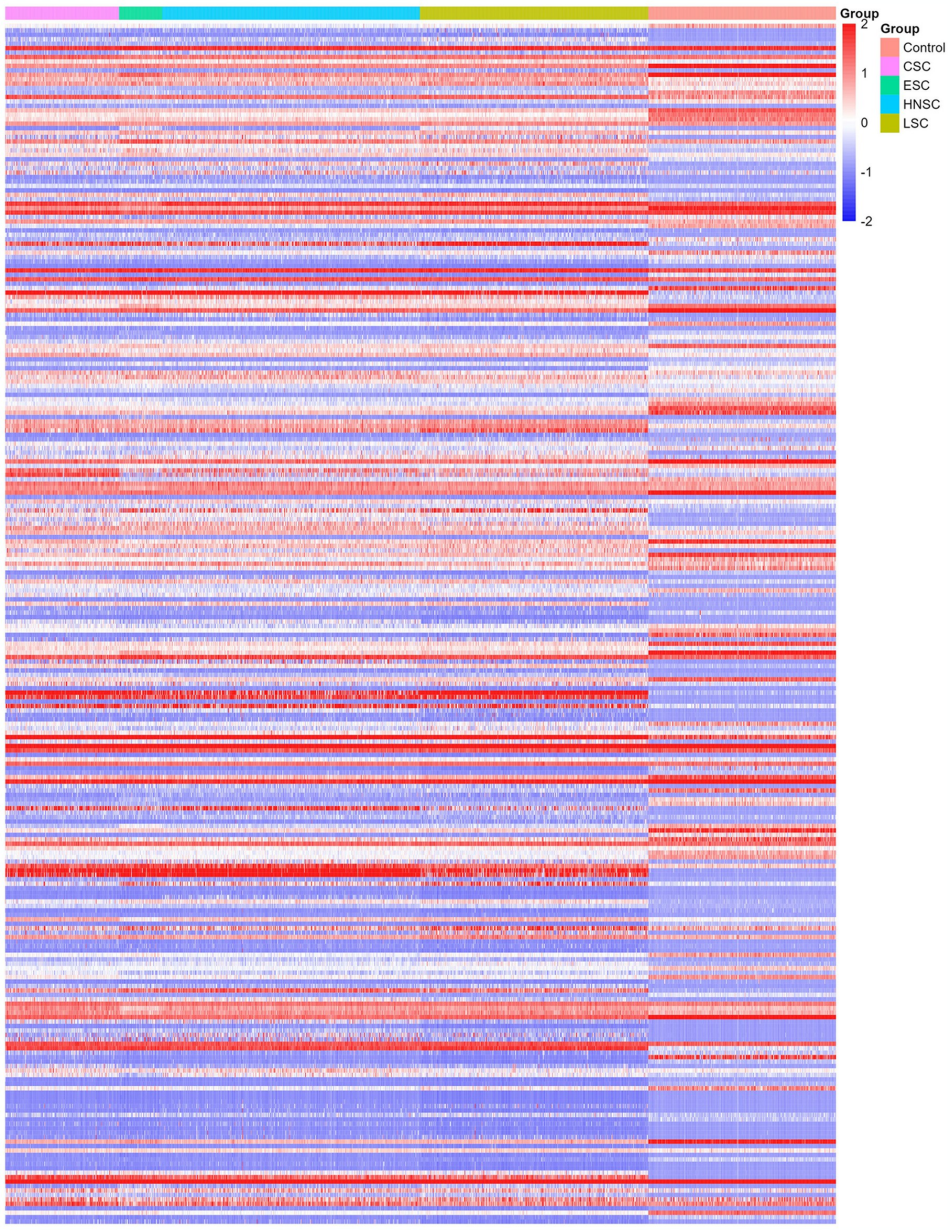
Heatmap plot of DEGs. Heatmap plot distinguishing the expression pattern of DEGs from SCC tumor tissues to adjacent normal tissues.

**Fig 8:**
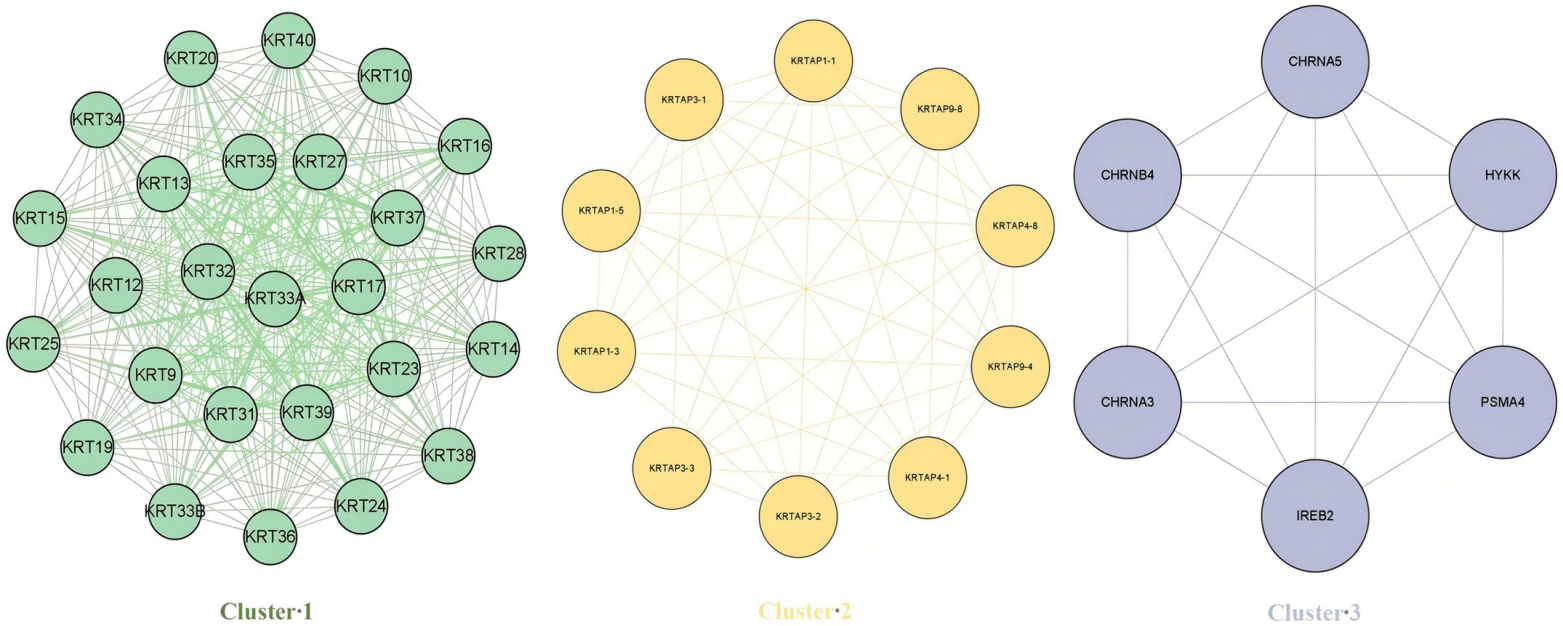
PPI analysis of DEGs. PPI analysis showing the molecular interactions among the DEGs.

Following quality control, normalization and batch effect adjustment, expression profiles of CRV-Genes from SCCs samples, including both tumor and adjacent normal tissues, were compared. Principal component analysis (PCA) demonstrated clear difference between SCC tumor tissues and adjacent normal tissues based on CRV-genes expression data (see S3 Fig). A total of 270 significant DEGs were identified out of 469 CRV-Genes analyzed, among which 178 were up regulated and 92 were down regulated (Fig 6). Hierarchical clustering analysis of these DEGs revealed distinct expression pattern between SCC tumor tissues and adjacent normal tissues (Fig 7). The 270 proteins encoded by the selected DEGs were analyzed to construct a PPI network, which comprised 270 nodes and 715 edges, indicating a highly interconnected network with a PPI enrichment p-value<1×10^−16^ and an average local clustering coefficient of 0.434. Through the application the Molecular Complex Detection Algorithm (MCODE), three significant modules from the PPI network complex were identified, showing the intricate molecular interactions among the DEGs in SCC patients (Fig 8). The functional annotation of these clusters revealed that these genes were mainly associated with the Keratin (KET) and keratin-associated protein (KETAP) family, consistent with the findings of the differential expression analysis that identified KRT-related genes were overexpressed in SCCs tumor tissues.

### Comparison the susceptibility of index SNPs to Pan-SCCs and Single-SCCs

**Fig 9:**
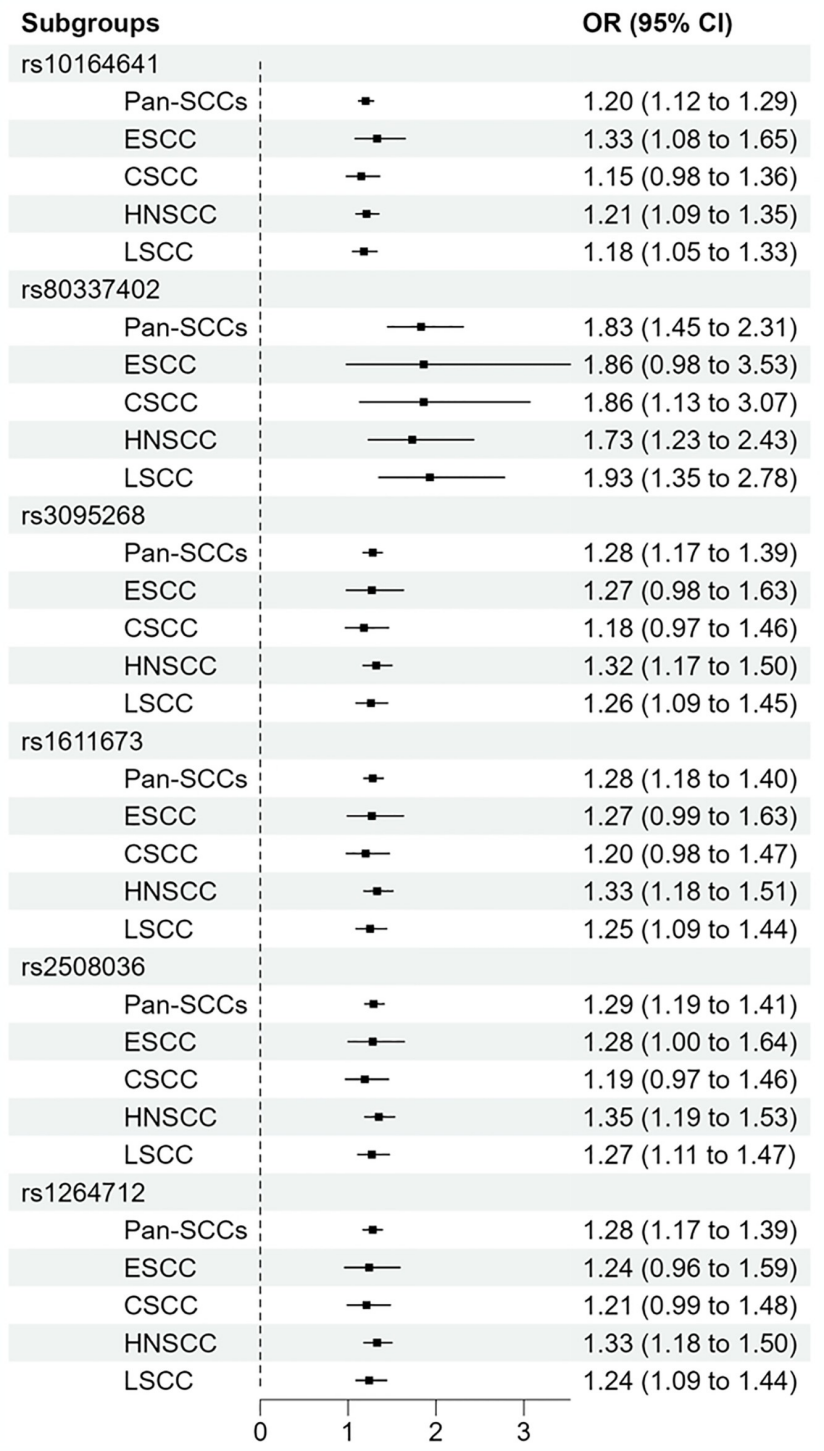
Index SNPs forest plots for Pan-SCCs versus Single-SCCs. In each study, positive results were represented by squares, and the 95% confidence interval was shown by horizontal bars.

The comparison of ORs between Pan-SCCs and Single-SCCs GWAS revealed that, although the index SNPs identified in Pan-SCCs study did not achieve genome-wide significance in the Single-SCCs GWAS, the direction of effect sizes was consistent across both Pan-SCCs and Single-SCCs GWAS. For example, rs10164641, identified as a risk SNP in the Pan-SCCs GWAS with an OR of 1.20 (95%CI 1.12–1.29), was also associated with increased risk of four types of SCCs in the Single-SCCs GWAS, with ORs ranging from 1.15 to 1.33. Notably, the ORs for each index SNPs did not significantly differ between the Pan-SCCs and Single-SCCs GWAS (Fig 9), indicating a uniform influence of these SNPs on SCC susceptibility across the aggregated and individual cancer types.

## Discussion

In the current study, a comprehensive genome-wide analysis combined with functional annotation was conducted to elucidate the biological significance of variants and genes associated with Pan-SCC susceptibility. Leveraging data of 1,928 Pan-SCC cases and 7,712 healthy controls from the UKB, we identified 6 novel loci associated with increased risk of Pan-SCC. Notably, all identified loci demonstrated significant interactions with fresh fruit intake, highlighting it as an important lifestyle factor influencing SCCs development. GO and KEGG pathway enrichment analyses underscored the critical role of immune-related genes and pathways in carcinogenesis of SCCs, with a special emphasis on the HLA region. Further analysis of HLA alleles and amino acids revealed significant associations with SCCs risk. Moreover, differential expression analysis, coupled with PPI network and modular analysis, indicated the pivotal role of KRT and KRTAP genes in SCCs pathology, suggesting these genes’ activities as potentially integral to the disease mechanism.

The primary distinction of our study from earlier GWAS on SCCs lies in our unique case selection approach, which involves treating SCCs cases across various sites collectively as a unified case group, in contrast to conventional approach that focuses exclusively on single-site SCCs. Our analysis based on Pan-SCCs cases underscores the multifactorial nature of SCCs, affirming that its development is influenced by both genetic predispositions and environmental factors, consistent with findings from single-site SCCs [25–28]. In line with previous research, we identified smoking, alcohol consumption, and reduced intake of vegetable and fruits as contributing risk factors for SCCs. Notably, our investigation into the gene-environment interactions revealed a significant multiplicative interaction specifically between fresh fruit intake and index SNPs, enriching the existing body of knowledge that has primarily emphasized the interactions between smoking, alcohol use, and genetic factors in increasing SCCs risk [28–30].

In the GWAS analysis, we identified six novel loci predominantly located within non-coding regions, with four specifically located in the HLA region. This finding, reinforced by enrichment analysis results, suggests a potential link between the HLA region SCCs susceptibility. HLA genes, critical for encoding cell-surface proteins responsible for presenting antigen peptides to the host immune system, are recognized for their extensive polymorphism, making them one of the most variable gene groups in the human genome [31]. A notable features of the HLA region is its strong and complex LD across long genetic distances [32]. While previous studies has implicated HLA alleles in the pathogenesis of SCCs, the direct linkage to specific causal variants has remained elusive [33–36]. In our study, by using SNP2HLA for the imputation of HLA genes, we are able to identify both classical HLA alleles and specific amino acid positions that potentially affect susceptibility, thus enhancing our understanding of the genetic landscape within the HLA region that contributes to SCCs risk [37].

Analysis of transcriptome data yielded 270 DEGs, prompting us to perform PPI network and modular analysis to identify risk genes. In particular, KRT and KRTAP gene families, known for their role in forming intermediate filaments in epithelial cells, emerged as potential significant contributors to the pathogenesis of SCCs. These genes are implicated in cancer cell invasion, metastasis, and drug resistance, serving as diagnostic and prognostic markers in epithelial cancers [38]. Our findings indicate an overexpression of KRT genes in SCCs tumor tissue, characterized by strong protein-protein interactions. This corroborates previous research linking the risk of SCCs to the abnormal expression of specific KRT genes (e.g., KRT1, KRT4, KRT17 and KRT19) [39–42]. Additionally, certain KRT genes show high specificity for SCCs, potentially distinguishing them from other cancer subtypes [43–45]. Despite this, our findings on enriched pathways are limited, underscoring the need for further research to elucidate the complex role of KRT genes in the progression of SCCs.

The major strength of this study is the focus on the Pan-SCC phenotype, moving beyond previous Single-SCC research to unveil consistent susceptibility effects and discover new risk loci. Additionally, employing a comprehensive multi-omics approach to analyze genomics and transcriptomics data has provided new insights into the biological mechanisms of SCCs. Furthermore, the study’s extension into gene-environment interactions and detailed functional analyses has substantially deepened our understanding of SCCs risk factors. Nevertheless, this study has some limitations. First, the findings largely rely on the UKB and TCGA databases, where significant variation in sample sizes across the four SCCs subtypes were noted, potentially introducing selection bias. Second, combining risk variants from only European and Chinese populations may obscure the genetic diversity among different ethnicities and individuals. Finally, for HLA analysis, the imputation accuracy dependents on the match between the target population and reference panel, and the available genotype data, limited to classical HLA types at four-digit resolution, may not fully capture the effect of the HLA region on SCCs.

## Conclusions

In conclusion, through the integration of multi-omics data, and both individual level and publicly available biological datasets, we have identified novel loci associated with SCCs susceptibility and illustrated their substantial interactions with fresh fruit intake. Further functional analyses provided novel insights into the roles of the HLA genes and KRT genes in SCCs, potentially offering new biological targets for prevention and treatment strategies.

## Supporting information

S1 Table The histological coding of SCCs included in the study. (DOCX)

S2 Table The susceptibility locus of SCCs. (DOCX)

S3 Table Association of index SNPs and SCCs risk. (DOCX)

S1 Fig Q-Q plot for the Pan-SCCs genome-wide association study. (DOCX)

S2 Fig Regional locus zoom plots of six index SNPs. (DOCX)

S3 Fig PCA plot of CRV-Genes. (DOCX)
