# Supplementary material for: Shared genetic factors and the interactions with fresh fruit intake contributes to four types squamous cell carcinomas
Source: PLoS One. 2024 Dec 31;19(12):e0316087. doi: 10.1371/journal.pone.0316087 (PMC11687899; doi:10.1371/journal.pone.0316087)
Supplement: S1 Table — (DOCX) [file pone.0316087.s001.docx]

S1 Table. The histological coding of SCCs included in the study.

| Coding | Description |
| --- | --- |
| 8052 | Papillary squamous cell carcinoma |
| 8053 | Squamous cell papilloma, inverted |
| 8070 | Squamous cell carcinoma |
| 8071 | Squamous cell carcinoma, keratinizing |
| 8072 | Squamous cell carcinoma, large cell, non-keratinizing |
| 8073 | Squamous cell carcinoma, small cell, non-keratinizing |
| 8074 | Squamous cell carcinoma, spindle cell |
| 8075 | Squamous cell carcinoma, adenoid |
| 8076 | Squamous cell carcinoma, micro-invasive |
| 8078 | Squamous cell carcinoma with horn formation |
| 8083 | Basaloid squamous cell carcinoma |
