# Supplementary material for: Shared genetic factors and the interactions with fresh fruit intake contributes to four types squamous cell carcinomas
Source: PLoS One. 2024 Dec 31;19(12):e0316087. doi: 10.1371/journal.pone.0316087 (PMC11687899; doi:10.1371/journal.pone.0316087)
Supplement: S2 Table — (DOCX) [file pone.0316087.s002.docx]

S2 Table. The susceptibility locus of SCCs. Summary of significant SNPs in the Pan-SCCs Genome-wide association study(sorted in ascending order by P value).

| CHROM | BP | SNP | OR | L95 | U95 | P |
| --- | --- | --- | --- | --- | --- | --- |
| 6 | 29923008 | rs2508036 | 1.29329 | 1.18646 | 1.40974 | 5.02E-09 |
| 6 | 29911222 | rs1059535 | 1.29398 | 1.18663 | 1.41103 | 5.45E-09 |
| 6 | 29920272 | rs1621510 | 1.29072 | 1.18437 | 1.40663 | 6.01E-09 |
| 6 | 29910663 | rs707910 | 1.2897 | 1.18351 | 1.40543 | 6.52E-09 |
| 6 | 29899015 | rs1655930 | 1.2895 | 1.18299 | 1.4056 | 7.46E-09 |
| 6 | 29939854 | rs369073 | 1.28627 | 1.18103 | 1.4009 | 7.46E-09 |
| 6 | 29910731 | rs79361534 | 1.28737 | 1.18119 | 1.40309 | 8.84E-09 |
| 6 | 29908676 | rs1632890 | 1.28729 | 1.18101 | 1.40315 | 9.26E-09 |
| 6 | 29907961 | rs439935 | 1.28622 | 1.18025 | 1.4017 | 9.59E-09 |
| 6 | 29842545 | rs2734963 | 1.28662 | 1.18045 | 1.40235 | 9.73E-09 |
| 6 | 29910910 | rs9260146 | 1.28603 | 1.18005 | 1.40152 | 9.86E-09 |
| 6 | 29913374 | rs1061815 | 1.28588 | 1.17996 | 1.40132 | 9.89E-09 |
| 6 | 29914696 | rs2571384 | 1.28588 | 1.17996 | 1.40132 | 9.89E-09 |
| 6 | 29916203 | rs1655899 | 1.28588 | 1.17996 | 1.40132 | 9.89E-09 |
| 6 | 29916618 | rs1655900 | 1.28588 | 1.17996 | 1.40132 | 9.89E-09 |
| 6 | 29909021 | rs2571420 | 1.28596 | 1.17999 | 1.40145 | 9.93E-09 |
| 6 | 29913137 | rs3132686 | 1.28482 | 1.17896 | 1.4002 | 1.12E-08 |
| 6 | 29906815 | rs401241 | 1.28419 | 1.17843 | 1.39944 | 1.17E-08 |
| 6 | 29918022 | rs1655905 | 1.2841 | 1.17829 | 1.39942 | 1.20E-08 |
| 6 | 29918059 | rs1632878 | 1.2841 | 1.17829 | 1.39942 | 1.20E-08 |
| 6 | 29837507 | rs885932 | 1.28623 | 1.17944 | 1.40268 | 1.25E-08 |
| 6 | 29825415 | rs1611673 | 1.28169 | 1.17605 | 1.39682 | 1.56E-08 |
| 6 | 29961274 | rs1632926 | 1.27858 | 1.17404 | 1.39241 | 1.63E-08 |
| 6 | 29818568 | rs2734986 | 1.28118 | 1.17555 | 1.3963 | 1.66E-08 |
| 6 | 29834805 | 6:29834805_A_G | 1.28114 | 1.1755 | 1.39628 | 1.68E-08 |
| 6 | 29958807 | rs1264807 | 1.27808 | 1.17358 | 1.39187 | 1.72E-08 |
| 6 | 29966787 | rs356959 | 1.27743 | 1.17312 | 1.39102 | 1.77E-08 |
| 6 | 29829592 | rs1611718 | 1.28036 | 1.17486 | 1.39535 | 1.78E-08 |
| 6 | 29827956 | rs1611701 | 1.28152 | 1.17555 | 1.39704 | 1.78E-08 |
| 6 | 29836463 | 6:29836463_C_T | 1.28026 | 1.1747 | 1.39531 | 1.83E-08 |
| 6 | 29836647 | 6:29836647_G_C | 1.28026 | 1.1747 | 1.39531 | 1.83E-08 |
| 6 | 29839672 | rs1611645 | 1.28026 | 1.1747 | 1.39531 | 1.83E-08 |
| 6 | 29840156 | rs1611647 | 1.28026 | 1.1747 | 1.39531 | 1.83E-08 |
| 6 | 29820002 | rs2508052 | 1.28006 | 1.17451 | 1.39509 | 1.87E-08 |
| 6 | 29825510 | 6:29825510_A_G | 1.27986 | 1.17436 | 1.39483 | 1.89E-08 |
| 6 | 29825219 | 6:29825219_AG_A | 1.27962 | 1.17421 | 1.39449 | 1.89E-08 |
| 6 | 29820448 | rs5013089 | 1.27962 | 1.17421 | 1.39449 | 1.89E-08 |
| 6 | 29828435 | rs1611703 | 1.27973 | 1.17421 | 1.39474 | 1.94E-08 |
| 6 | 29821937 | rs2517862 | 1.27934 | 1.17394 | 1.39419 | 1.95E-08 |
| 6 | 29820951 | rs2734983 | 1.2793 | 1.17391 | 1.39415 | 1.96E-08 |
| 6 | 29821032 | rs2517866 | 1.2793 | 1.17391 | 1.39415 | 1.96E-08 |
| 6 | 29821837 | rs9258636 | 1.2793 | 1.17391 | 1.39415 | 1.96E-08 |
| 6 | 29822070 | rs9258642 | 1.2793 | 1.17391 | 1.39415 | 1.96E-08 |
| 6 | 29822415 | rs9258652 | 1.2793 | 1.17391 | 1.39415 | 1.96E-08 |
| 6 | 29822649 | rs2517857 | 1.2793 | 1.17391 | 1.39415 | 1.96E-08 |
| 6 | 29822659 | rs2517856 | 1.2793 | 1.17391 | 1.39415 | 1.96E-08 |
| 6 | 29823164 | rs2517855 | 1.2793 | 1.17391 | 1.39415 | 1.96E-08 |
| 6 | 29823883 | 6:29823883_A_G | 1.2793 | 1.17391 | 1.39415 | 1.96E-08 |
| 6 | 29824183 | 6:29824183_T_C | 1.2793 | 1.17391 | 1.39415 | 1.96E-08 |
| 6 | 29824771 | 6:29824771_G_A | 1.2793 | 1.17391 | 1.39415 | 1.96E-08 |
| 6 | 29826726 | 6:29826726_T_A | 1.2793 | 1.17391 | 1.39415 | 1.96E-08 |
| 6 | 29826907 | 6:29826907_G_A | 1.2793 | 1.17391 | 1.39415 | 1.96E-08 |
| 6 | 29827149 | 6:29827149_C_T | 1.2793 | 1.17391 | 1.39415 | 1.96E-08 |
| 6 | 29827332 | 6:29827332_C_T | 1.2793 | 1.17391 | 1.39415 | 1.96E-08 |
| 6 | 29827597 | rs1611698 | 1.2793 | 1.17391 | 1.39415 | 1.96E-08 |
| 6 | 29828622 | rs1611706 | 1.2793 | 1.17391 | 1.39415 | 1.96E-08 |
| 6 | 29829407 | rs1611714 | 1.2793 | 1.17391 | 1.39415 | 1.96E-08 |
| 6 | 29829514 | rs1611716 | 1.2793 | 1.17391 | 1.39415 | 1.96E-08 |
| 6 | 29830209 | rs1611721 | 1.2793 | 1.17391 | 1.39415 | 1.96E-08 |
| 6 | 29830670 | rs1611725 | 1.2793 | 1.17391 | 1.39415 | 1.96E-08 |
| 6 | 29830725 | rs1611727 | 1.2793 | 1.17391 | 1.39415 | 1.96E-08 |
| 6 | 29831418 | rs1611736 | 1.2793 | 1.17391 | 1.39415 | 1.96E-08 |
| 6 | 29831706 | rs1611738 | 1.2793 | 1.17391 | 1.39415 | 1.96E-08 |
| 6 | 29831814 | rs1611740 | 1.2793 | 1.17391 | 1.39415 | 1.96E-08 |
| 6 | 30023079 | rs9261259 | 1.27639 | 1.1721 | 1.38996 | 2.01E-08 |
| 6 | 29821270 | rs3094176 | 1.27594 | 1.17181 | 1.38932 | 2.02E-08 |
| 6 | 29832692 | rs1611746 | 1.27873 | 1.17338 | 1.39353 | 2.08E-08 |
| 6 | 29832840 | rs2734977 | 1.27873 | 1.17338 | 1.39353 | 2.08E-08 |
| 6 | 29818726 | rs2523761 | 1.27592 | 1.17164 | 1.38948 | 2.13E-08 |
| 6 | 29832479 | rs1611744 | 1.27823 | 1.17292 | 1.393 | 2.19E-08 |
| 6 | 29832516 | rs1611745 | 1.27823 | 1.17292 | 1.393 | 2.19E-08 |
| 6 | 30052942 | rs1264712 | 1.27524 | 1.17107 | 1.38866 | 2.24E-08 |
| 6 | 29842451 | rs1611658 | 1.27657 | 1.17162 | 1.39093 | 2.43E-08 |
| 6 | 29988642 | rs3129012 | 1.27435 | 1.17025 | 1.38771 | 2.46E-08 |
| 6 | 29854631 | 6:29854631_CG_C | 1.27991 | 1.17352 | 1.39594 | 2.49E-08 |
| 6 | 29834472 | 6:29834472_C_T | 1.2771 | 1.17183 | 1.39182 | 2.51E-08 |
| 6 | 30056476 | rs1264709 | 1.2747 | 1.17038 | 1.38832 | 2.53E-08 |
| 6 | 30042209 | rs1264714 | 1.27382 | 1.1698 | 1.3871 | 2.58E-08 |
| 6 | 29832721 | 6:29832721_TC_T | 1.2768 | 1.17143 | 1.39166 | 2.70E-08 |
| 6 | 29832723 | rs150961114 | 1.2768 | 1.17143 | 1.39166 | 2.70E-08 |
| 6 | 29820182 | rs9258608 | 1.27335 | 1.1692 | 1.38677 | 2.85E-08 |
| 6 | 29991867 | rs259926 | 1.27262 | 1.16869 | 1.3858 | 2.92E-08 |
| 6 | 29979797 | rs356971 | 1.27188 | 1.16805 | 1.38496 | 3.12E-08 |
| 6 | 29606761 | rs3095268 | 1.27614 | 1.16974 | 1.39222 | 4.03E-08 |
| 6 | 30049966 | rs1150732 | 1.26879 | 1.16483 | 1.38204 | 4.83E-08 |
| 6 | 29753348 | rs9258381 | 1.25958 | 1.15838 | 1.36962 | 6.65E-08 |
| 6 | 29764472 | rs1633005 | 1.25933 | 1.15819 | 1.36931 | 6.75E-08 |
| 6 | 29771621 | rs1610660 | 1.26474 | 1.16101 | 1.37775 | 7.50E-08 |
| 6 | 29740477 | rs1610638 | 1.26177 | 1.1584 | 1.37436 | 9.73E-08 |
| 6 | 29742003 | rs1610639 | 1.2607 | 1.1574 | 1.37321 | 1.09E-07 |
| 4 | 185079351 | rs80337402 | 1.83428 | 1.45183 | 2.31748 | 3.68E-07 |
| 2 | 55123380 | rs10164641 | 1.20384 | 1.12011 | 1.29384 | 4.57E-07 |
| 6 | 30025503 | rs259919 | 1.2089 | 1.12276 | 1.30166 | 4.91E-07 |
