# Supplementary material for: Shared genetic factors and the interactions with fresh fruit intake contributes to four types squamous cell carcinomas
Source: PLoS One. 2024 Dec 31;19(12):e0316087. doi: 10.1371/journal.pone.0316087 (PMC11687899; doi:10.1371/journal.pone.0316087)
Supplement: S3 Table — (DOCX) [file pone.0316087.s003.docx]

S3 Table. Association of index SNPs and SCCs risk. The relationships between the six index SNPs and SCCs were evaluated among different genetic models, including codominant model, dominant model, and recessive model.

| **SNP** | **Model type** | **Genotype** | **Case(%)** | **Control(%)** | **X^2^** | **OR** | **P** |
| --- | --- | --- | --- | --- | --- | --- | --- |
| **rs10164641** | Codominant | TT | 562(30.5) | 2589(35.0) | 25.231 | 1.00 | <0.001 |
|  |  | CT | 890(48.3) | 3564(48.2) |  | 1.15(1.02,1.29) |  |
|  |  | CC | 389(21.1) | 1234(16.7) |  | 1.45(1.25,1.68) |  |
|  | Dominant | TT | 562(30.5) | 2589(35.0) | 13.397 | 1.00 | <0.001 |
|  |  | CT+CC | 1279(69.5) | 4798(65.0) |  | 1.23（1.10，1.37） |  |
|  | Recessive | CT+TT | 1452(78.9) | 6153(83.3) | 19.907 | 1.00 | <0.001 |
|  |  | CC | 389(21.1) | 1234(16.7) |  | 1.34（1.18，1.52） |  |
| **rs80337402** | Codominant | TT | 1736(94.3) | 7161(96.9) | 31.058 | 1.00 | <0.001 |
|  |  | CT | 105(5.7) | 224(3.0) |  | 1.93(1.52,2.45) |  |
|  |  | CC | 0(0.0) | 2(0.0) |  | NA(0.00,NA) |  |
|  | Dominant | TT | 1736(94.3) | 7161(96.9) | 29.791 | 1.00 | <0.001 |
|  |  | CT+CC | 105(5.7) | 226(3.1) |  | 1.92(1.51,2.43) |  |
|  | Recessive | TT+CT | 1841(100) | 7385(100) | / | 1.00 | <0.001 |
|  |  | CC | 0(0.0) | 2(0.0) |  | NA(0.00,NA) |  |
| **rs3095268** | Codominant | GG | 1130(61.4) | 5040(68.2) | 32.325 | 1.00 | <0.001 |
|  |  | GA | 619(33.6) | 2076(28.1) |  | 1.33(1.19,1.49) |  |
|  |  | AA | 92(5.0) | 271(3.7) |  | 1.51(1.18,1.93) |  |
|  | Dominant | GG | 1130(61.4) | 5040(68.2) | 31.194 | 1.00 | <0.001 |
|  |  | AG+AA | 711(38.6) | 2347(31.8) |  | 1.35(1.22,1.50) |  |
|  | Recessive | GG+AG | 1749(95.0) | 7116(96.3) | 6.8847 | 1.00 | <0.05 |
|  |  | AA | 92(5.0) | 271(3.7) |  | 1.38(1.08,1.76) |  |
| **rs1611673** | Codominant | AA | 4943(66.9) | 1102(59.9) | 34.969 | 1.00 | <0.001 |
|  |  | AG | 2160(29.2) | 638(34.7) |  | 1.32(1.19,1.48) |  |
|  |  | GG | 284(3.8) | 101(5.5) |  | 1.60(1.26,2.02) |  |
|  | Dominant | AA | 4943(66.9) | 1102(59.9) | 32.473 | 1.00 | <0.001 |
|  |  | AG+GG | 2444(33.1) | 739(40.1) |  | 1.36(1.22,1.51) |  |
|  | Recessive | AA+AG | 1740(94.5) | 7103(96.2) | 9.933 | 1.00 | <0.01 |
|  |  | GG | 101(5.5) | 284(3.8) |  | 1.45(1.15,1.83) |  |
| **rs2508036** | Codominant | GG | 1101(59.8) | 4952(67.0) | 36.201 | 1.00 | <0.001 |
|  |  | GC | 640(34.8) | 2151(29.1) |  | 1.34(1.20,1.49) |  |
|  |  | CC | 100(5.4) | 284(3.8) |  | 1.58(1.35,2.01) |  |
|  | Dominant | GG | 1101(59.8) | 4952(67.0) | 34.155 | 1.00 | <0.001 |
|  |  | GC+CC | 740(40.2) | 2435(33.0) |  | 1.37(1.23,1.52) |  |
|  | Recessive | GG+GC | 1741(94.6) | 7103(96.2) | 9.3097 | 1.00 | <0.01 |
|  |  | CC | 100(5.4) | 284(3.8) |  | 1.44(1.14,1.82) |  |
| **rs1264712** | Codominant | GG | 1081(58.7) | 4857(65.8) | 33.263 | 1.00 | <0.001 |
|  |  | GA | 657(35.7) | 2227(30.1) |  | 1.33(1.19,1.48) |  |
|  |  | AA | 103(5.6) | 303(4.1) |  | 1.53(1.21,1.93) |  |
|  | Dominant | GG | 1081(58.7) | 4857(65.8) | 31.77 | 1.00 | <0.001 |
|  |  | GA+AA | 760(41.3) | 2530(34.2) |  | 1.35(1.22,1.5) |  |
|  | Recessive | GG+GA | 1738(94.4) | 7084(95.9) | 7.8099 | 1.00 | <0.01 |
|  |  | AA | 103(5.6) | 303(4.1) |  | 1.39(1.1,1.74) |  |
