# Supplementary material for: Shared genetic factors and the interactions with fresh fruit intake contributes to four types squamous cell carcinomas
Source: PLoS One. 2024 Dec 31;19(12):e0316087. doi: 10.1371/journal.pone.0316087 (PMC11687899; doi:10.1371/journal.pone.0316087)

S1 Fig. Q-Q plot for the Pan-SCCs Genome-wide association study. The genomic inflation factor (λ) was estimated to be 1.032, suggesting no systematic inflation in the GWAS.


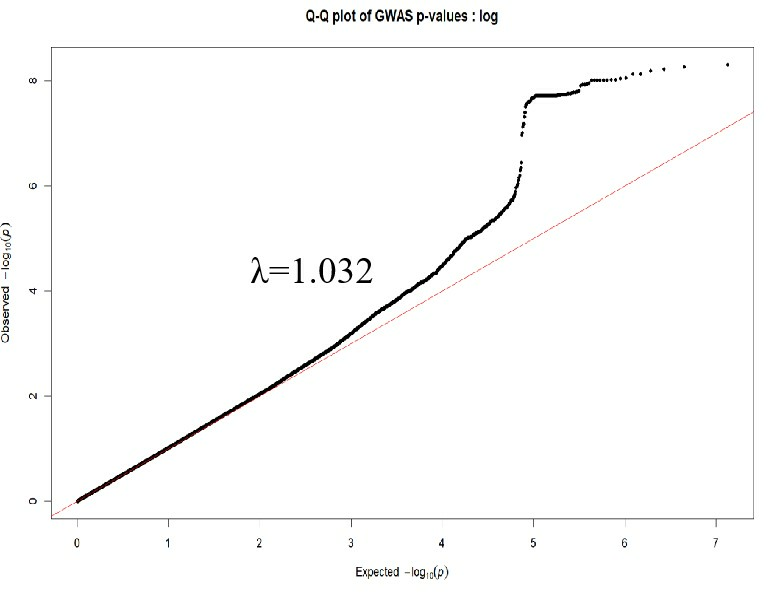

Supplement: S1 Fig — (DOCX) [file pone.0316087.s004.docx]
