# Supplementary material for: Shared genetic factors and the interactions with fresh fruit intake contributes to four types squamous cell carcinomas
Source: PLoS One. 2024 Dec 31;19(12):e0316087. doi: 10.1371/journal.pone.0316087 (PMC11687899; doi:10.1371/journal.pone.0316087)

S2 Fig. Regional locus zoom plots of six index SNPs. The SNPs surrounding each index SNP are color coded to reflect their correlation. Each dot is colored by r2 of linkage disequilibrium (LD) with the purple-colored index SNP indicated with texts (chromosome position). Genes, the position of exons and direction of transcription from UCSC genome browser are noted. Plots were generated using LocusZoom.


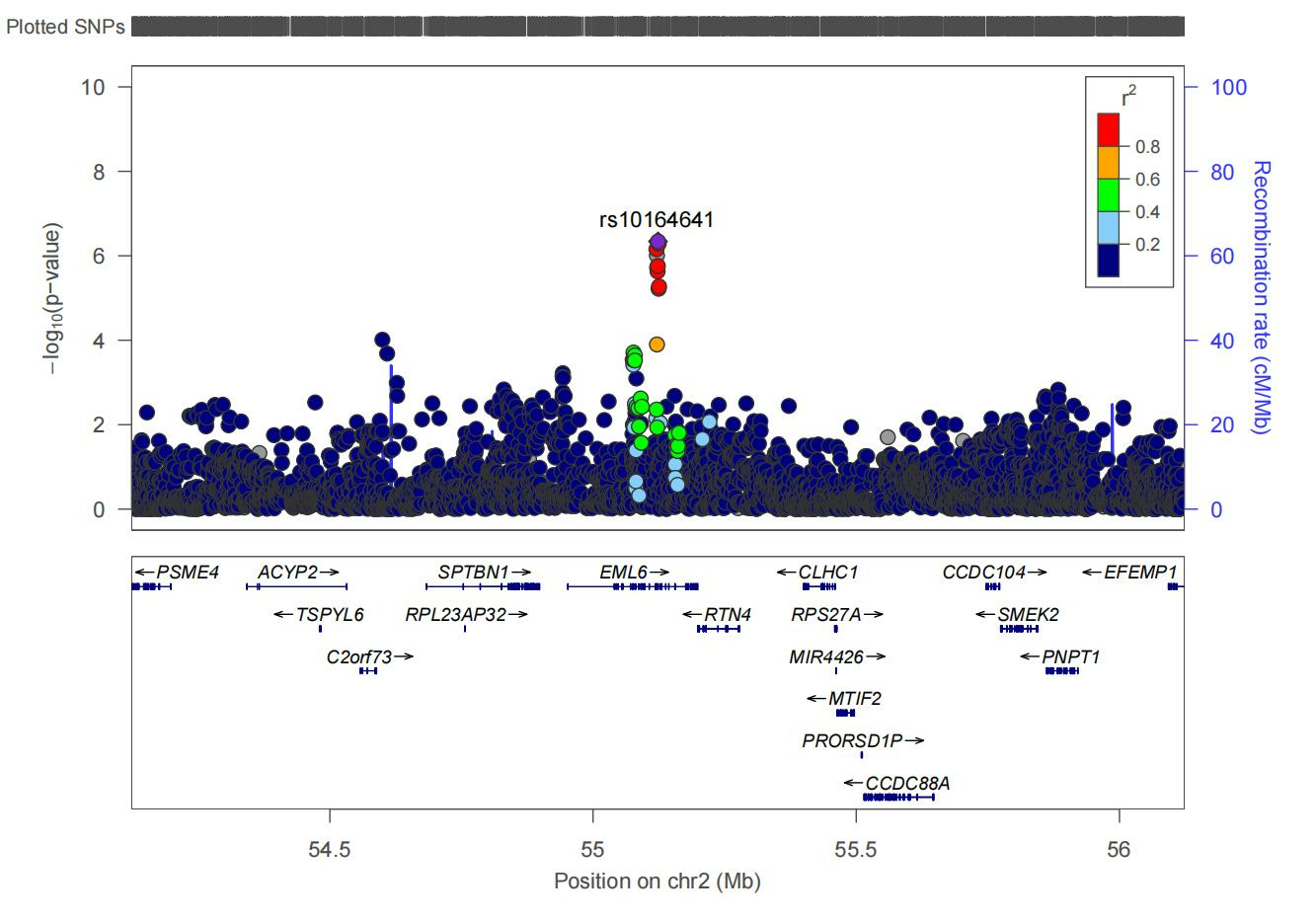

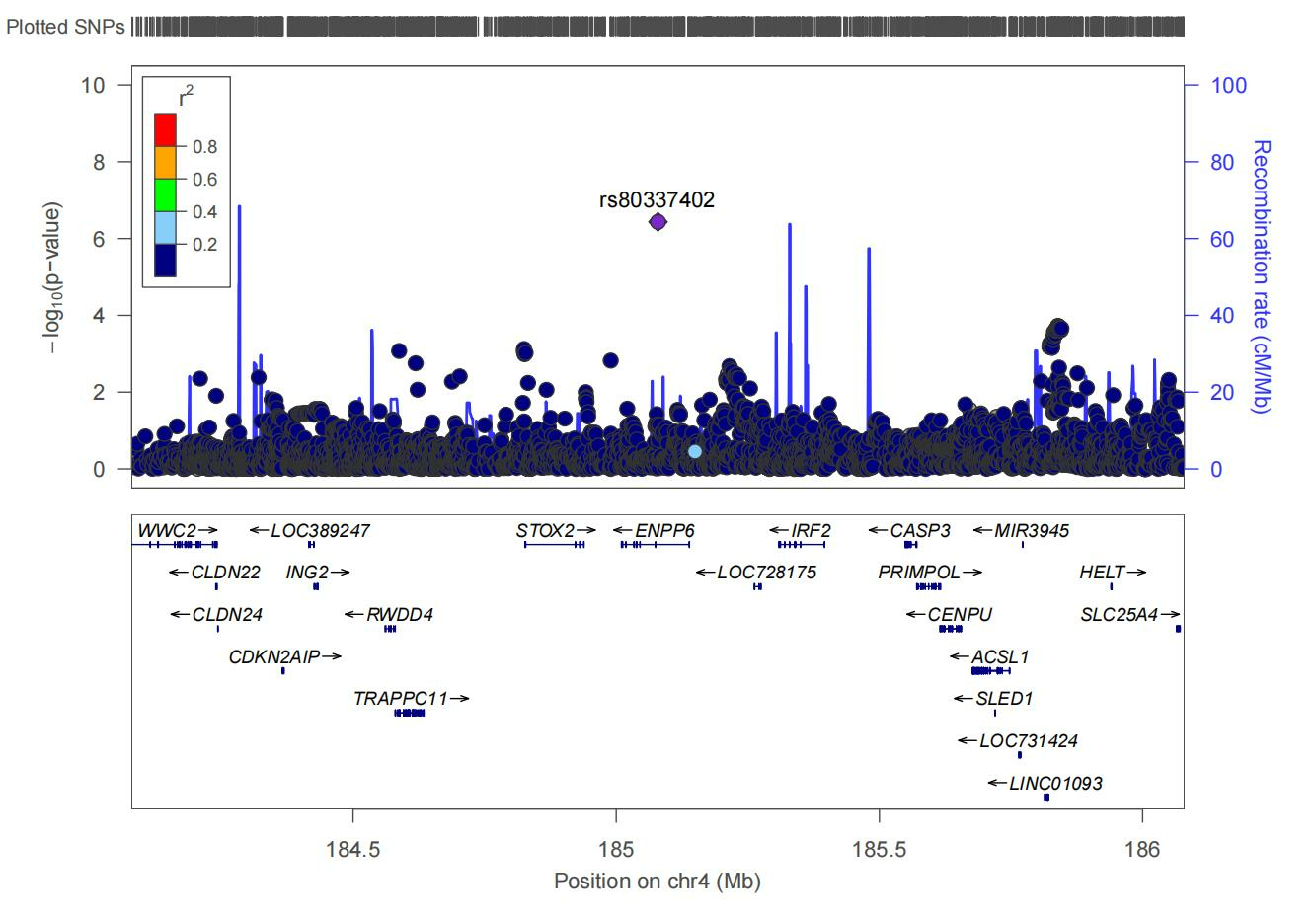


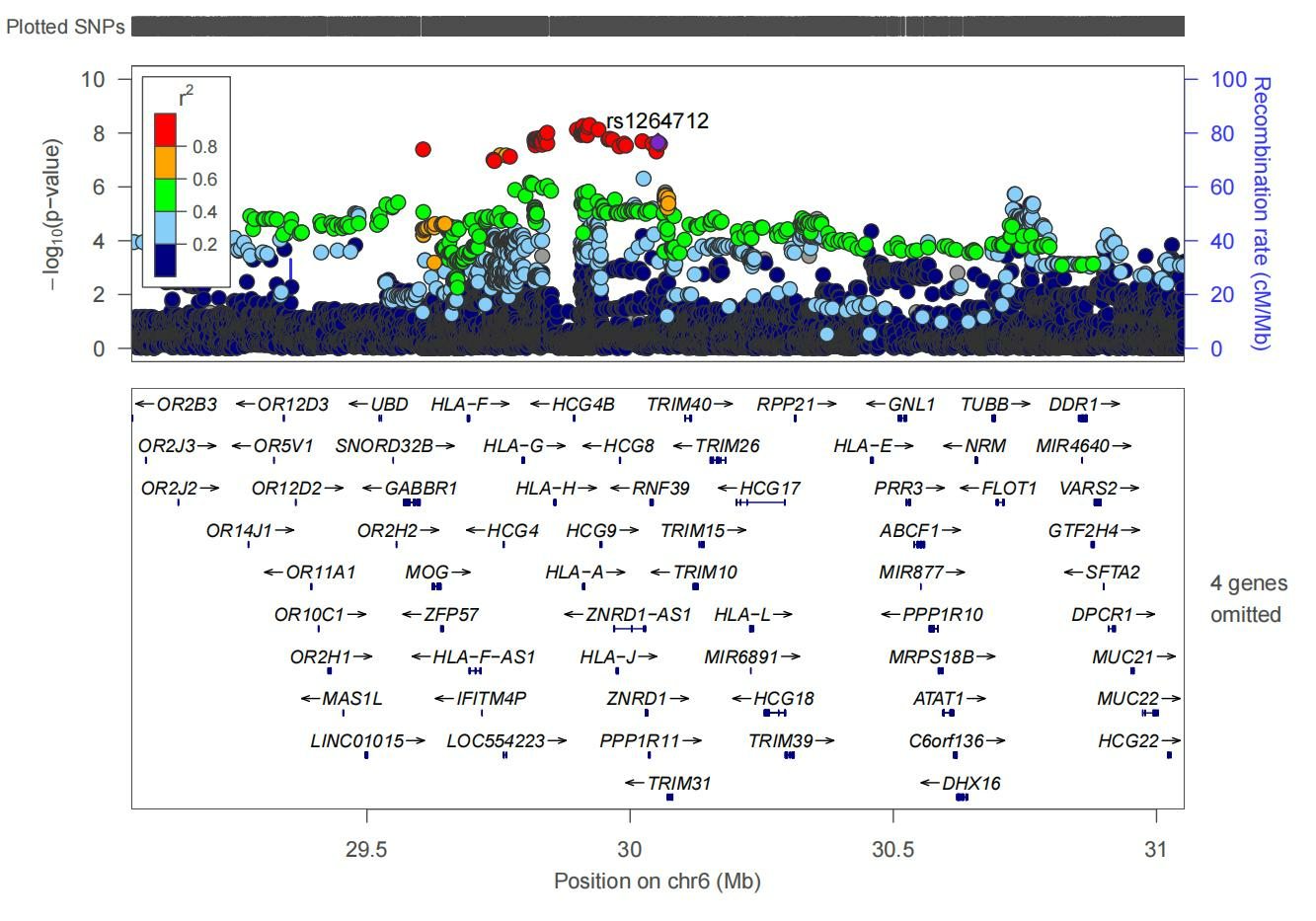


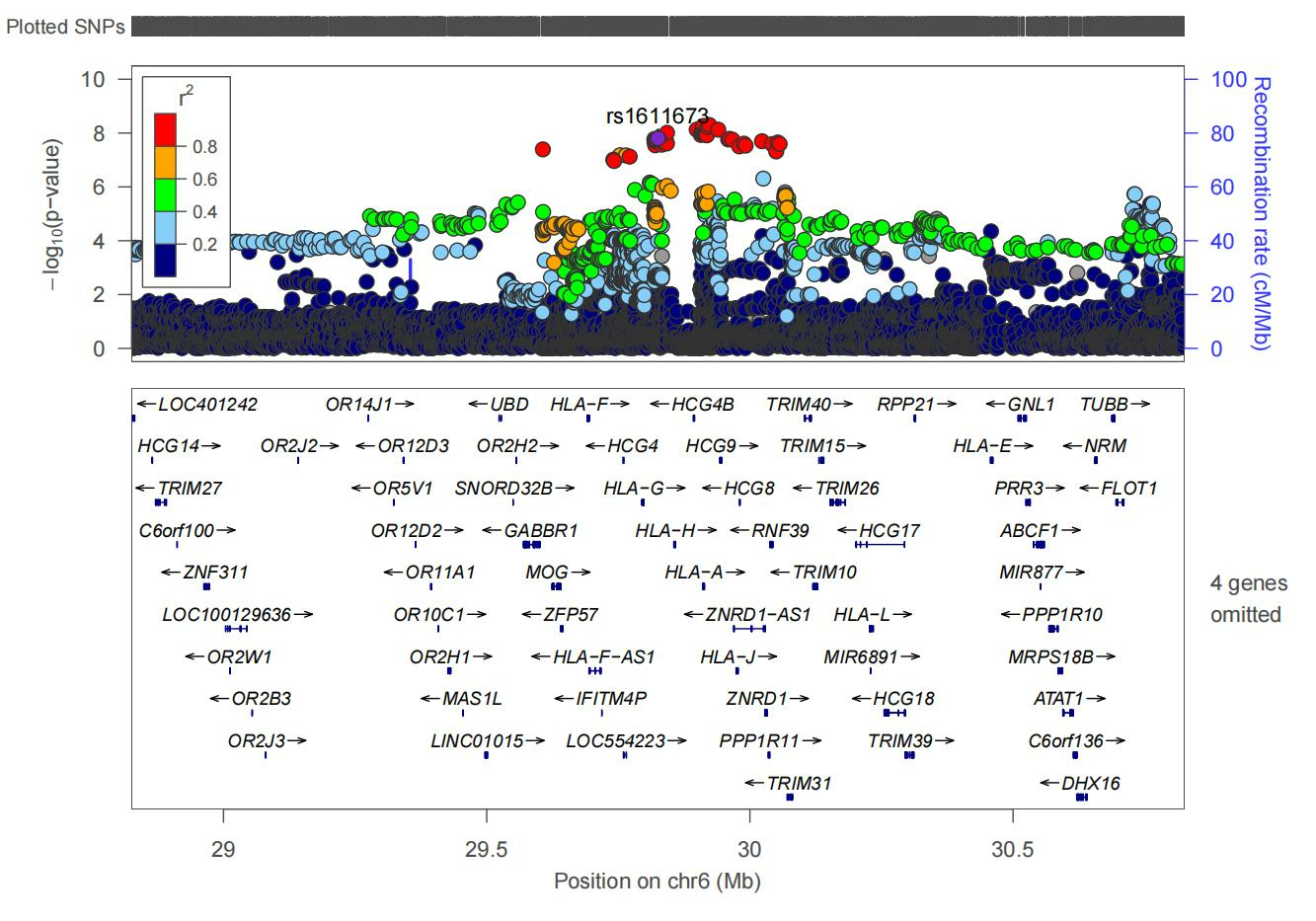


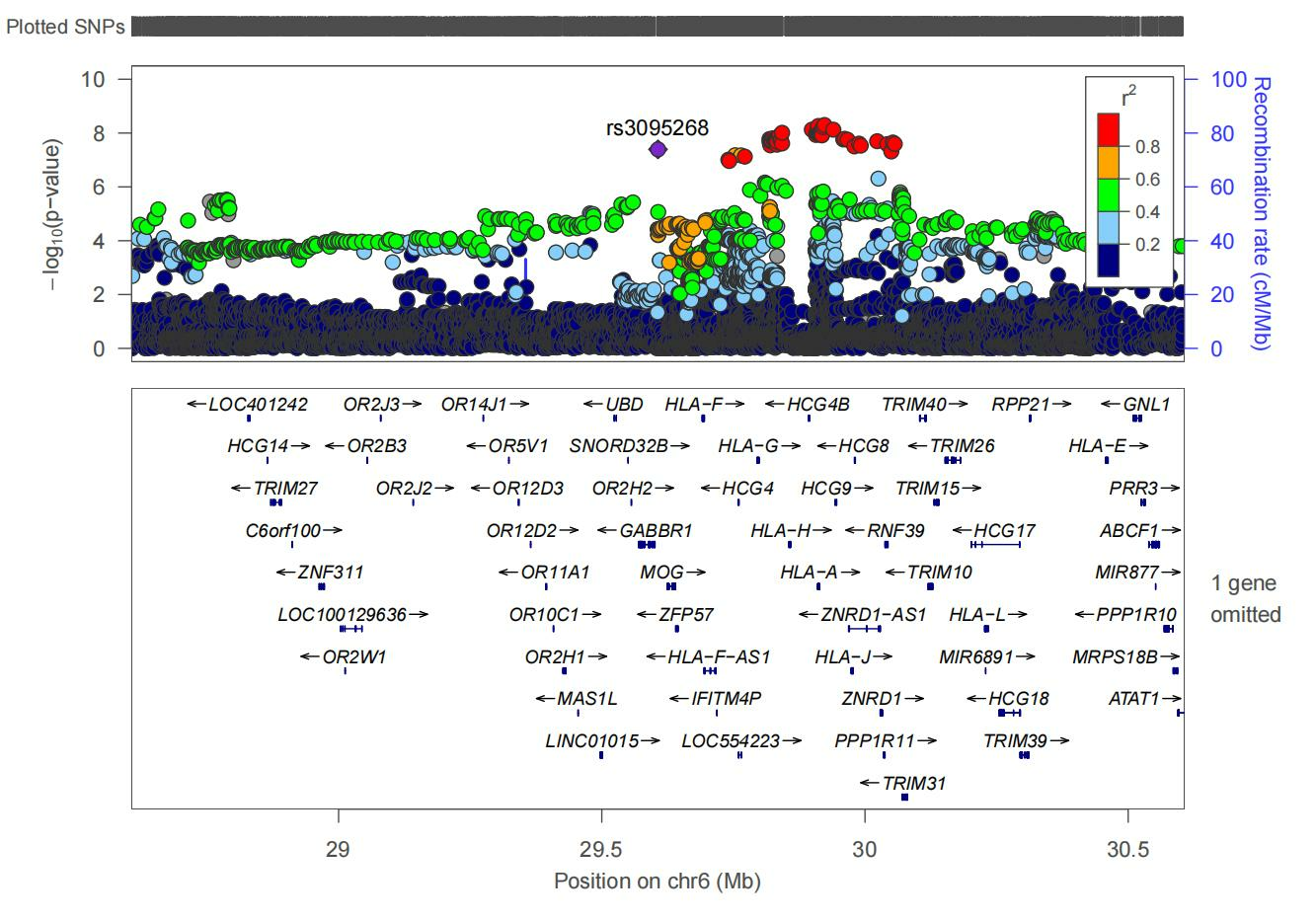

Supplement: S2 Fig — (DOCX) [file pone.0316087.s005.docx]
