# Supplementary material for: Shared genetic factors and the interactions with fresh fruit intake contributes to four types squamous cell carcinomas
Source: PLoS One. 2024 Dec 31;19(12):e0316087. doi: 10.1371/journal.pone.0316087 (PMC11687899; doi:10.1371/journal.pone.0316087)

S3 Fig. PCA plot of CRV-Genes. Principal component analysis based on the expression data of CRV-Genes indicated two significantly distinct patterns.


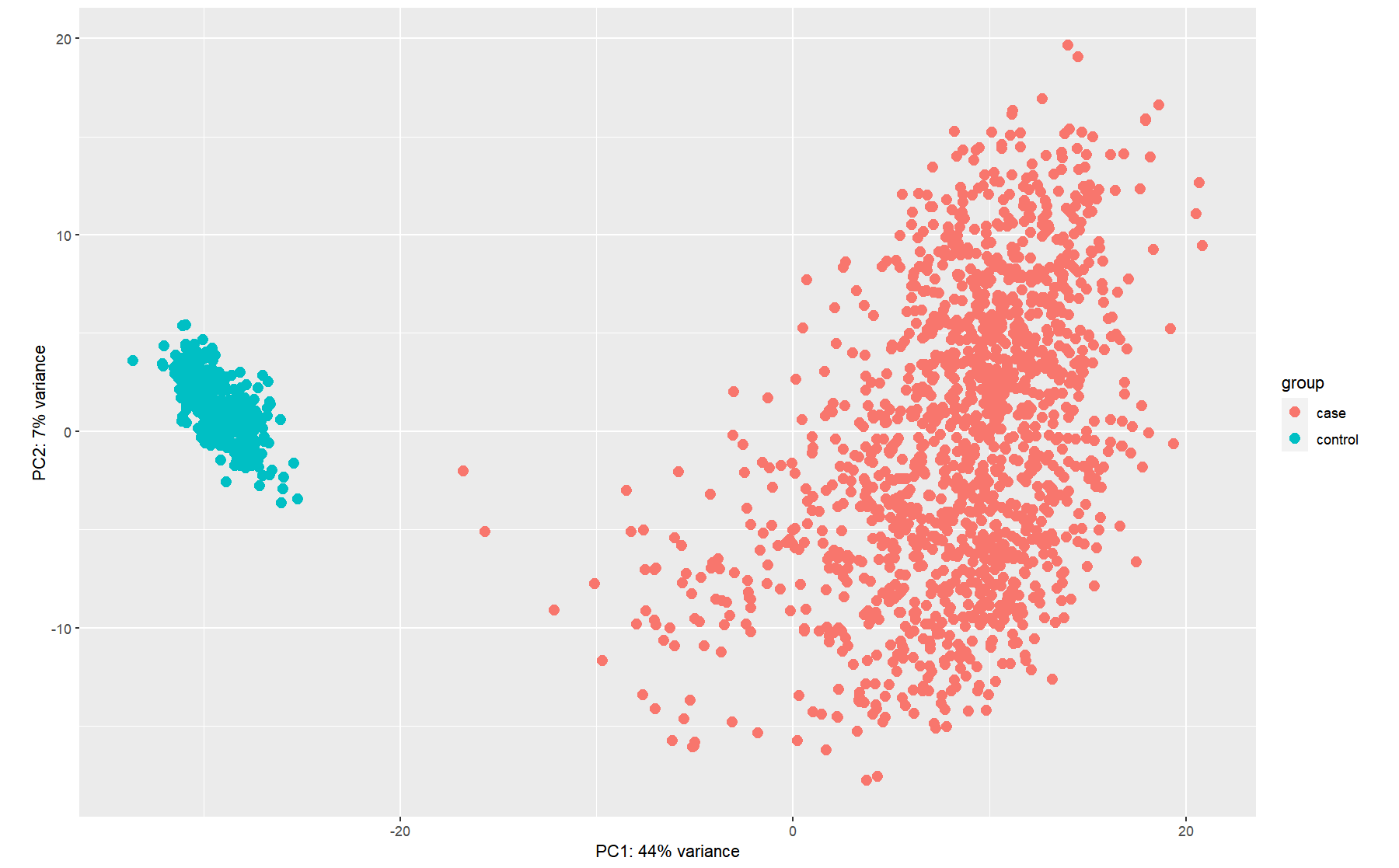

Supplement: S3 Fig — (DOCX) [file pone.0316087.s006.docx]
